# Stabilization and Carbonization of Electrospun Organosolv Lignin Fibers from *Miscanthus x giganteus* to Produce Biobased Carbon Fiber

**DOI:** 10.3390/polym18202496

**Published:** 2026-10-12

**Authors:** Roland Jacks Ekila, Tatjana Stevanovic, Denis Rodrigue

**Affiliations:** 1 Department of Wood Sciences, Faculty of Forestry, Laval University, Quebec, QC G1V 0A6, Canada; roland-jacks.ekila.1@ulaval.ca; 2 Department of Chemical Engineering, Laval University, Quebec, QC G1V 0A6, Canada; denis.rodrigue@gch.ulaval.ca

**Keywords:** lignin, *Miscanthus x giganteus*, stabilization, carbon fibers

## Abstract

This paper focuses on the stabilization and carbonization of fiber mats produced by electrospinning organosolv lignin derived from *Miscanthus x giganteus*, with no additives. Stabilization and carbonization were carried out to obtain electrospun lignin fiber mats. The stabilization step was carried out under air at a heating rate of 0.25 °C/min to maximum temperatures of between 200 and 300 °C. Then, the carbonization step was performed under nitrogen at a heating rate of 5 °C/min to maximum temperatures of 800 and 1000 °C and durations of 1 and 2 h. TGA, DSC, SEM–EDS, XPS, FTIR, Raman, and XRD analyses were carried out to determine the specific characteristics of the stabilized and carbonized material obtained. The DSC analysis showed that the fibers were well stabilized, which resulted in an increase in the glass transition temperature (Tg) compared to the starting material. SEM analysis showed an overall 35% decrease in the average fiber diameter during the stabilization and carbonization processes. The results of the Raman spectroscopy showed an ID/IG ratio of about 1 for all the carbon fibers, which indicates that the material tends to graphitize. X-ray diffraction analysis of the stabilized and carbonized fibers revealed a spectral peak around 23° for all samples and demonstrated a partially ordered (turbostratic) state of the material. Finally, ultra-thin carbon fibers with a carbon content of 97% were obtained after carbonization at 1000 °C.

## 1. Introduction

Carbon fiber (CF) represents a highly advanced technological material for today’s industry, with a promising future for the development of numerous sectors (transport, aeronautics, electrodes, sporting equipment and others) [1,2,3,4,5]. Carbon fiber is characterized by its high strength (tensile strength of 0.5–13 GPa, tensile modulus of 35–1800 GPa) and low weight (density of 1.75–1.95 g/cm^3^) [2,6,7,8]. Industrial CF is five times stronger than steel and twice as stiff, with a lower density [1,2,9,10,11].

Based on a high-carbon-content material, the success of carbon fiber processing depends on stabilization and carbonization procedures that not only prevent the fibers from melting but also facilitate the removal of the majority of non-carbon atoms [9,12,13]. Produced primarily from petrochemical precursor materials (polyacrylonitrile and pitch), industrial carbon fibers have environmental limitations [2,5,6]. The production of CF, of petrochemical origin, presents both environmental problems, linked to CO_2_ emissions, and economic problems due to the high and unstable cost of the resources [3,5,14,15]. According to [12], the complex preparation process and the high production cost of PAN-based CF limit their application and development to some extent.

For an alternative that is both ecological and economical, lignocellulosic materials (aromatic lignin biopolymers in particular) present great potential for the development of an eco-responsible CF [12,14,16,17,18,19]. Lignins are three-dimensional aromatic (polyphenolic) polymers made up of p-hydroxyphenyl, guaiacyl, and syringyl units, linked by β–O–4, β–β, β–5, 5–5 bonds [19,20]. They are considered as promising CF precursors because of their aromatic structure (high content of *sp*^2^ hybridized carbons), which promotes graphitization during lignin fiber carbonization [21].

Lignin also offers the advantage of being a renewable resource at low cost, with a high carbon content (above 60%) [19,22,23]. However, various industrial lignins produced from pulping processes of lignocellulosic biomass present uniformity problems, making them unsuitable for CF production; this is mainly due to their low molecular mass and high polydispersity, which leads to very fragile lignin-based fibers [19]. Industrial lignins currently benefit from limited valorization beyond energy and chemical recovery in kraft processes. However, it is estimated that more intensive lignins’ use could reduce the cost of CF production by 37 to 49% [2,7,12,24].

Lignins are commonly transformed into CF by electrospinning from lignin solutions, generating fibers of different diameters [25,26]. Among the various lignin spinning techniques, electrospinning is highly valued for its simplicity, versatility, and capacity to produce a material with a very small diameter [26,27,28,29,30]. Lignin fibers obtained by electrospinning from various solutions and mixtures with synthetic polymers are generally transformed by two heating processes, as described above [21,31]. These highly energy-intensive steps require controlled high-temperature environments and consume 200 to 300 MJ of energy per kg of fiber produced [5]. According to [14], lignin-based materials are generally fragile and cannot be easily handled.

Therefore, the majority of studies performed so far used mixtures/blends with synthetic polymers as plasticizers [14]. With or without additives, one of the biggest challenges for lignin-based CF concerns the stabilization and carbonization steps [3,9,13,32,33]. Although lignin inherently contains fixed carbon, a stabilization step is essential to permanently solidify the electrospun fibers before carbonization at higher temperatures [9]. Lignin contains an aromatic cyclic structure that contributes to the stabilization of its structure during high-temperature processes [16]. Stabilization is applied to prevent the melting of lignin fibers. It is commonly performed in the presence of air in a temperature range of 200–300 °C, with heating rates of 0.1–2 °C/min [13]. Stabilization affects the glass transition temperature (Tg), preventing softening during heat treatments to produce CF [31].

The stabilization step promotes the dehydrogenation and cyclization reactions of the macromolecular lignin structures, which convert lignin fibers into a heat-resistant trapezoidal structure, thus producing a thermally stable polymer structures through the integration of oxygenated functional groups, among other reactions [9,12,13]. During stabilization, hydroxyl groups participate in radical reactions, leading to a more condensed, crosslinked and stable structure [3,32].

During stabilization, the fibers exhibit a core-shell structure that forms on the surface and prevents oxygen from penetrating the deeper layers, thus ensuring that only minor changes occur in the central part of the lignin fiber [33]. Stabilization also prevents the melting and adhesion of fibers between them during the subsequent carbonization step [12]. However, an excessively high stabilization temperature can lead to fiber shrinkage, weakening the tension-induced lignin reorganization, thus preserving more oxygen bonds and reducing the process of graphite structure formation [9].

Carbonization is commonly carried out in an inert (nitrogen) atmosphere at high temperatures (600–3000 °C). These conditions allow for the conversion of the stabilized polymer material into a predominantly carbonaceous material at a faster heating rate (5–10 °C/min), ultimately leading to graphitic structure formation [13,33]. The carbonization process of lignin-based fibers involves heat and mass transfer due to the high temperature, resulting in the removal of non-carbon elements from the fibers, while reducing the fiber diameter [34]. As carbonization progresses, the levels of residual methoxy, carbonyl, and carboxyl groups decrease, while the ratio of fused to unfused aryl carbons increases [33]. When lignin is carbonized, it turns into different carbons with various microcrystalline structures, depending on the type of starting lignin used [35].

*Miscanthus x giganteus*, a herbaceous plant, has already been used for nanofiber development [17]. Reference [36] showed the feasibility of producing nanofibers from *Miscanthus x giganteus* cellulose. Indeed, Yang et al. [36] successfully produced cellulose nanofibers functionalized with carboxylic acid groups, isolated from a sustainable hybrid grass, *Miscanthus x giganteus*, using a novel two-step chemical oxidation process comprising bleach treatment and ammonium persulfate oxidation, followed by ultrasonication. *Miscanthus x giganteus* is at the heart of an ambitious project by the Canadian startup DMT Bioproduct, which aims to process the plant for the production of bioproducts. The decision to use it for developing lignin-based carbon fibers stems from the quantity and structure of its lignin. *Miscanthus x giganteus* contains approximately 25% lignin, with a composition comprising p-hydroxyphenyl, guaiacyl, and syringyl units [26].

Soda-like lignin from Miscanthus was electrospun from a dimethylformamide (DMF) solution with 50 wt.% PAN to obtain CF [17]. *Miscanthus x giganteus* is unique because it is a very energetic plant with a fairly high level of carbon fixation [37,38,39].

The organosolv lignin obtained from *Miscanthus x giganteus* contains p-hydroxyphenyl, guaiacyl and syringyl aromatic groups. Its high carbon content (above 70%) may be an advantage, despite its low molecular weight (around 1000 g/mol) [26,40]. Beyond the advantages of lignin-based materials, most approaches rely on complex activation processes, metallic additives or multi-step treatments, which increase costs and reduce the possibility of scaling up [23].

The novelty of this study resides in the fact that a dimethyl sulfoxide (DMSO) solution of lignin is proposed for the production of electrospun lignin fiber mats with no polymer addition, followed by their further stabilization and carbonization. In particular, organosolv lignin derived from *Miscanthus x giganteus* was solubilized in DMSO and then electrospun without additives [26], in order to preserve the completely biobased nature of the final product. The stabilization and carbonization steps were also carried out through process optimization without any additives.

## 2. Experimental Approach

The methodology used in this study involves the air stabilization and nitrogen carbonization of electrospun lignin fibers produced as described in our previous study [26]. This study consists of two parts: optimization of the stabilization and carbonization steps of electrospun lignin fiber mats and the characterization of the materials obtained.

### 2.1. Materials

This study uses electrospun lignin fibers obtained in a previous study (fiber mat) with a thickness of around 5 mm and an average fiber diameter of 2 µm. The lignin fiber mat was obtained by electrospinning a lignin-DMSO solution at a concentration of 63 wt.% [26].

### 2.2. Methods

The electrospun lignin fibers were air-dried for 2 weeks before proceeding to stabilization under air. Once the fiber mat was stabilized, the second step followed the carbon fiber manufacturing process. Fiber stabilization was performed using a Thermo Scientific Lindberg/Blue M muffle furnace (Waltham, MA, USA). It was carried out in the presence of air at a heating rate of 0.25 °C/min with maximum temperatures of 200, 250, and 300 °C for a duration of 1 or 2 h.

After stabilization, the carbonization of the stabilized fibers was performed in an OTF-1200X tubular furnace from MTI Corp (Richmond, CA, USA). Carbonization was carried out under a nitrogen atmosphere at maximum temperatures of 800 or 1000 °C for durations of 1 or 2 h, at a heating rate of 5 °C/min. The stabilization process, carried out at 200, 250, and 300, was performed repeatedly, with 50 trials per temperature. Carbonization was also repeated, with an average of 20 trials per experimental condition. The lignin fiber mats were produced using a repetitive electrospinning process under identical conditions. On average, 200 electrospinning experiments were conducted to produce the lignin fiber mats.

### 2.3. Characterization of the Stabilized and Carbonized Fibers

The stabilized, carbonized fibers produced from electrospun lignin fiber mats were analyzed using different analytical methods, as described next. The lignin used to manufacture the fibers lignin had a purity of 89.30%; the impurities consisted of 6.65% residual sugars, 2% ash (minerals), and approximately 1% nitrogen. The average molecular weight of the lignin was determined to be 1040 g/mol, and the number molecular mass was 437 g/mol [27].

#### 2.3.1. Thermal Analyses

##### Differential Scanning Calorimetry (DSC)

DSC analysis was performed to determine changes in the lignins’ glass transition temperature during stabilization. The analyses were performed under a nitrogen atmosphere on Miscanthus organosolv lignin, electrospun lignin fiber, and stabilized fibers (obtained at 200, 250, and 300 °C) using a differential scanning calorimeter DSC 882e (Mettler Toledo, Columbus, OH, USA). DSC measurements were carried out at the same heating rate of 20 °C/min between 0 and 200 °C.

##### Thermogravimetric Analysis (TGA)

A thermogravimetric analysis (TGA) was performed to determine the stabilization and carbonization yields of the treated fibers. The instrument used for this analysis was a TGA/SDTA851 (Mettler Toledo, Columbus, OH, USA), operated under an air atmosphere for stabilization and a nitrogen atmosphere for carbonization, with each gas flowing at 50 mL/min. Stabilization was carried out between 35 and 300 °C at a heating rate of 0.25 °C/min, with a 2 h isotherm. Carbonization was performed between 35 and 1000 °C at a heating rate of 5 °C/min with 1 or 2 h isotherms.

#### 2.3.2. Spectroscopic Analyses

##### ATR-FTIR

FTIR analyses were carried out to determine the functional groups present according to changes in the treated materials. The spectra were acquired using a Perkin Elmer (Shanghai, China) spectrophotometer 400 in the attenuated total reflection (ATR) mode. Germanium crystals were used and all measurements were performed between 4000 and 400 cm^−1^, with a resolution of 2 cm^−1^ over 32 scan.

##### Raman

Raman spectra were obtained using a 532 nm laser to determine the crystalline state of the stabilized and carbon fibers. The equipment used was a SENTERRA II Raman microscope (Bruker Optics Inc., Billerica, MA, USA); a total of 5 accumulations of 1 s each were performed at a laser power of 25 mW in the 50–4000 cm^−1^ range using a ×20 microscope objective. × The microcrystalline size (La) of the stabilized and carbonized fibers was calculated from the relationship: La = 4.4/R, where R = I_D_/I_G_, with I_D_ being the intensity of the D-band peak and I_G_ being the intensity of the G-band peak [11].

#### 2.3.3. Scanning Electron Microscopy with Energy-Dispersive X-Ray Spectroscopy (SEM–EDS)

SEM–EDS analyses were performed on both stabilized fibers and carbon fibers. These analyses were carried out using a JEOL (Tokyo, Japan) JSM-6360LV scanning electron microscope (SEM) coupled with energy-dispersive X-ray spectroscopy (EDS) to determine the quality and composition of the obtained fibers. The diameter distribution of the stabilized and carbon fibers was determined using the Image J software version 1.53d. Images were randomly selected and a minimum of 50 fibers per type were used for statistical analyses.

#### 2.3.4. Chemical Properties of Fiber Surfaces Studied by X-Ray Photoelectron Spectroscopy (XPS)

The chemical composition of the surface of stabilized and carbon fibers was investigated by X-ray photoelectron spectroscopy (XPS) using a PHI 5600-ci spectrometer (Physical Electronics, Eden Prairie, MN, USA). The main XPS chamber was maintained at low pressure (less than 1 × 10^−8^ Torr). A standard aluminum X-ray source was used to record scanning and high-resolution (HR) spectra. Scanning analyses were performed with charge neutralization, while HR analyses were performed without charge neutralization. The detection angle was set at 45° to the surface normal, and the analyzed area was 0.05 cm^2^. The HR spectra curves were fitted using a least-squares minimization procedure based on Gaussian–Lorentzian functions and a continuous Shirley background. The C1s peaks were referenced at 285 eV (C-C and C-H).

#### 2.3.5. X-Ray Diffraction (XRD) of the Crystalline Properties of Fiber Samples

For XRD analysis, we used a Malvern Panalytical Empyrean X-ray diffractometer (Worcestershire, UK); during this analysis, a laser beam was directed at the sample using a 100× 0.75, producing a laser spot with a diameter of less than 2 µm. X-ray diffraction (XRD) with Cu Kα radiation (λ = 1.54 Å) enabled the structural characterization of the samples.

To determine the structural state of the stabilized and carbon-containing fibers, an evaluation of the crystallite size (Lc, nm) and the interplanar spacing (d, nm) was performed using Scherrer’s equations and Bragg’s law [3,11]:

(1) $$L_{c}=\;\frac{K\lambda \;}{\beta cos\theta }$$

(2) $$d=\frac{\lambda }{2\;sin\theta }$$

where:

*Lc*: grain size,

*d*: interplanar spacing,

*k*: Scherrer’s constant (0.94),

*λ*: X-ray wavelength (0.16 nm),

*β*: full width at half maximum (FWHM) of the diffraction peak,

*θ*: Bragg’s diffraction angle (23°).

## 3. Results and Discussion

### 3.1. Changes During Fiber Stabilization

The stabilization conditions applied made it possible to produce stable lignin fibers without melting. DSC analysis was performed to compare the thermal properties of organosolv lignin, electrospun lignin fibers and stabilized lignin fibers to determine any changes in the glass transition temperatures of the samples during the transformation.

**Figure 1:**
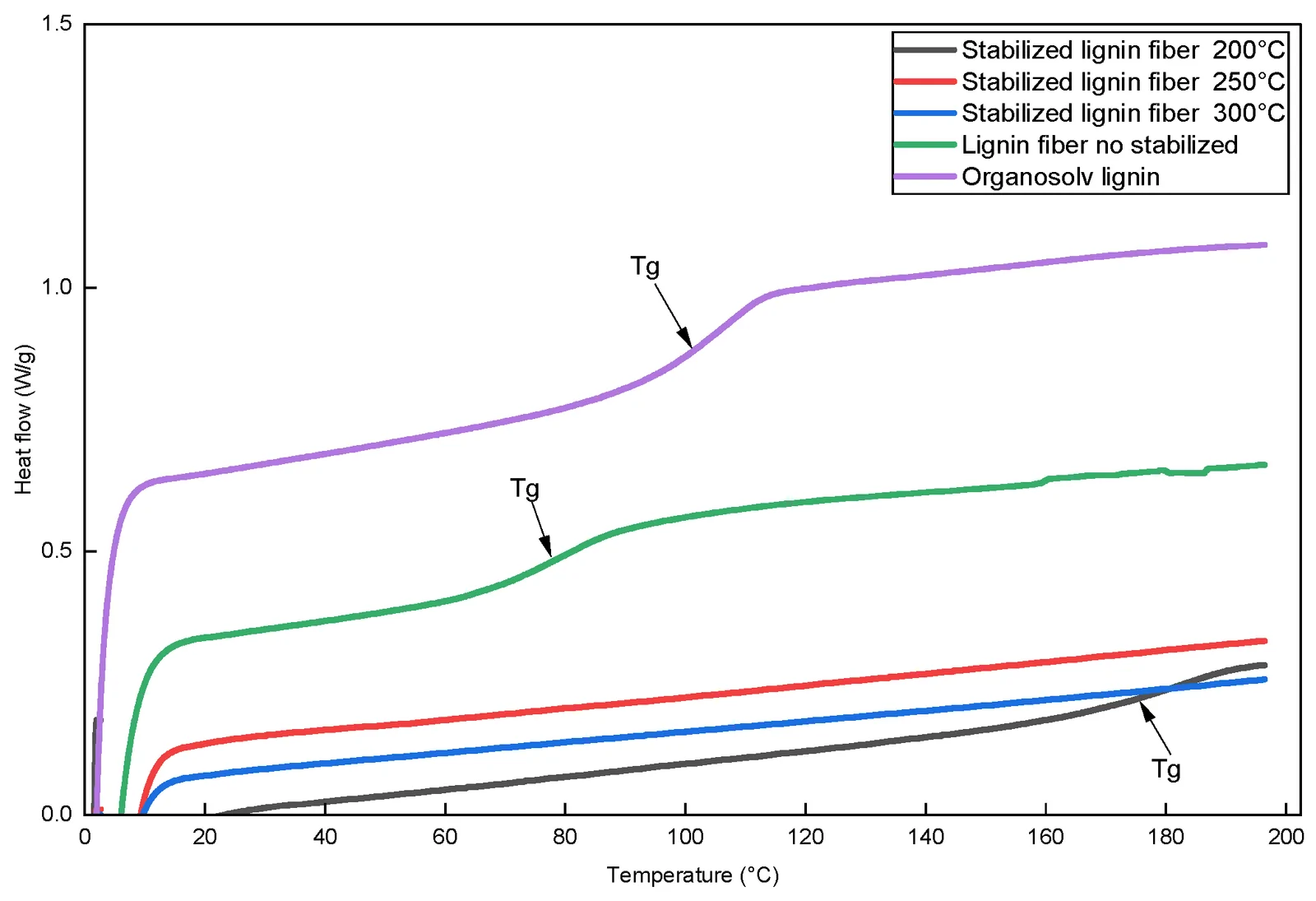
DSC results for Miscanthus organosolv lignin, lignin fibers, and stabilized lignin fibers under different conditions.

In Figure 1, a bump in the curve, illustrating the evolution of the glass transition temperature as a function of heat flux, is observed around 100 °C for organosolv lignin. This bump corresponds to the Tg of organosolv lignin derived from *Miscanthus x giganteus*. The lignin fiber evolution curve under the same conditions also shows a bump around 75 °C, confirming the presence of Tg for the stabilized lignin fiber, which is nonetheless lower than the value determined for the original lignin.

In the DSC curbs of stabilized lignin fibers, the positive shift of the inflection point indicates an increase in the glass transition temperature, demonstrating the fibers’ ability to withstand higher temperatures. An increase in Tg is observed during the stabilization process performed at 200 °C as the Tg value increases from 75 to 180 °C. For electrospun lignin fibers stabilized at temperatures above 200 °C, the Tg increased, indicating effective stabilization of the lignin fibers. This increase in Tg is related to crosslinking in the stabilized fibers, making them susceptible to further carbonization [7,23,25,41].

The stabilization of organosolv lignin fibers from *Miscanthus x giganteus* was thus achieved without any additives by optimizing the operating parameters. The favorable stabilization of *Miscanthus x giganteus* lignin fibers can be explained by changes in their structure. According to [15], the presence of a high number of syringyl (S) units compared to guaiacyl (G) units contributes to the improvement in the effectiveness of lignin thermal transformation. *Miscanthus x giganteus* lignin, which is rich in syringyl units, promotes an increase in Tg during the stabilization process [40]. Reference [42] also reported that the relative concentration of syringyl units is the main factor driving the variation of lignins’ Tg.

The stabilization time, including the isothermal hold (20 h), is roughly double the average time required to stabilize lignin fibers according to the literature [5,13]. Improving the stabilization time is essential because, among the three aspects of using alternatives to PAN, the alternative must have favorable processing characteristics, especially a shorter stabilization time. In general, the stabilization time for PAN is only around 5 min [10,43].

Depending on the temperature conditions used, the samples analyzed were stabilized at a low heating rate (0.25 °C/min) because melting was observed at higher heating rates. According to [32], to increase Tg and prevent softening during heat treatment aimed at producing carbonization of lignin fibers, an oxidative stabilization treatment at low heating rates prior to carbonization is necessary, which promotes polymer crosslinking reactions. The heating rate was one of the key parameters for achieving the stabilized state of the studied lignin fibers.

Besides the heating rate, another essential parameter for the successful stabilization of lignin fibers is the time spent at the maximum temperature. In this study, this time was extended to 2 h. Longer treatment times at similar temperatures are reported to promote crosslinking reactions and an increase in the Tg of the fibers [9]. However, longer treatment times and higher stabilization temperatures lead to fiber shrinkage during stabilization and reduce the ability of amorphous carbon to form graphitic carbon, thus reducing the crystal size and increasing crystal defects [9]. For these reasons, the operating conditions must be carefully selected for the stabilization of lignin fibers.

Thermogravimetric analysis (TGA) was performed on lignin fibers stabilized at 250 °C and for a duration of 2 h, although stabilization was possible between 200 and 300 °C. The aim of stabilizing the fibers between 200 and 300 °C was to validate data from the literature citing this temperature range for the stabilization processes of fibers intended for carbonization. Above 200 °C, we surpassed a Tg of over 160 °C, which was the highest glass transition temperature determined for the stabilized lignin fibers. Fibers stabilized at 250 °C were chosen to avoid an increase in the stabilization temperature, which could lead to defects during the cyclization of the aromatic rings According to [22], the high heat generated by the cyclization reaction is probably responsible for the defects observed during the formation of stabilized fiber. At 200 °C, the Tg value (180 °C) did not appear sufficient for the carbonization temperatures considered.

**Figure 2:**
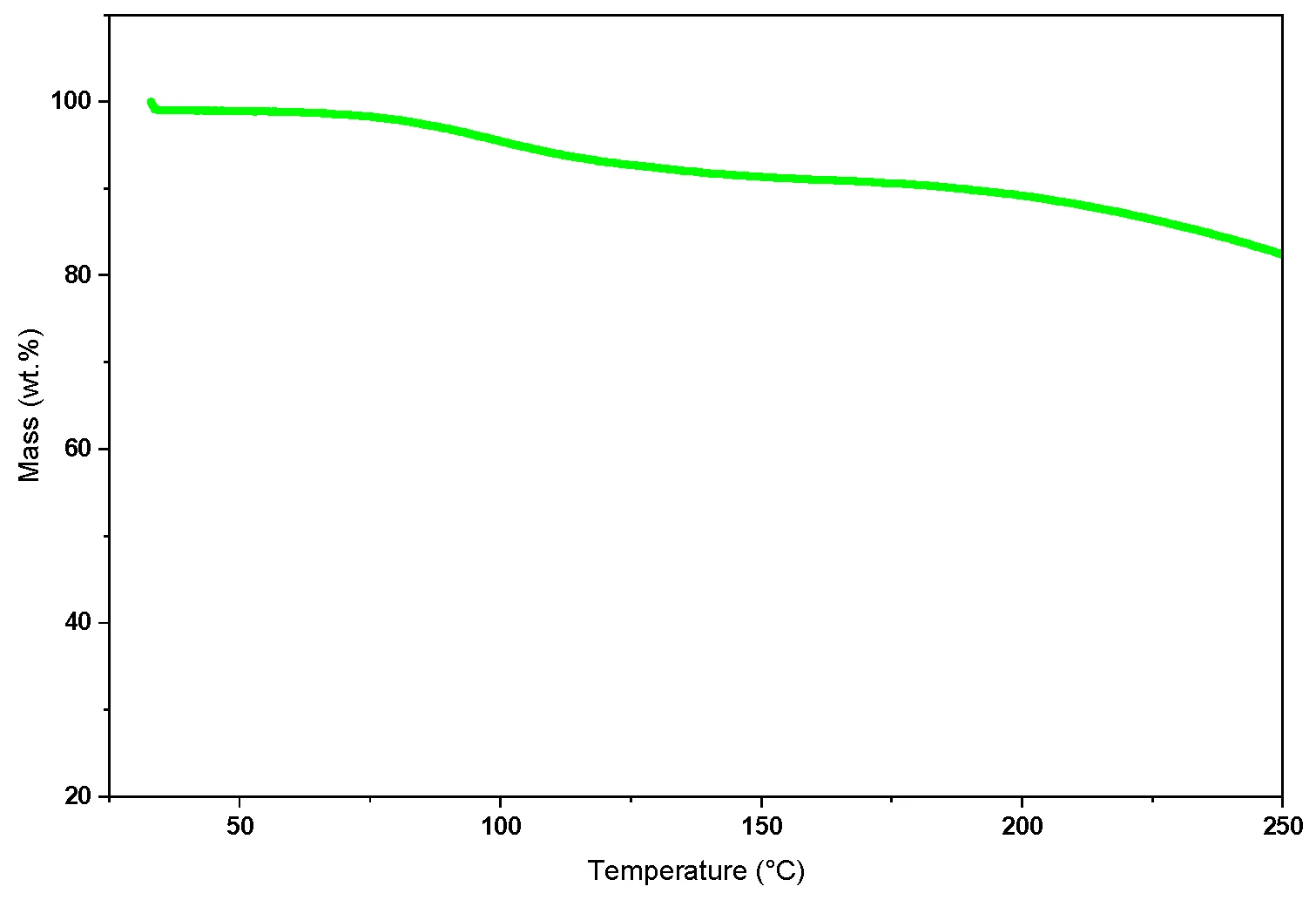
TGA results of lignin fibers stabilized at 250 °C.

Thermogravimetric analysis (TGA, Figure 2) of the studied lignin fibers shows a loss of material starting at 75 °C, which corresponds to the glass transition temperature (Tg) of the lignin fibers, as shown in Figure 1. A decrease in the curve, with a slope of 18%, is observed between 75 and 250 °C. This decrease is not pronounced; it is the result of optimizing the stabilization operating conditions.

The material loss described by TGA is related to both the evaporation of solvents, notably, water and dimethyl sulfoxide, and the elimination of hydroxide compounds [44]. Between 100 and 250 °C, the loss of material stabilizes due to higher Tg (Figure 1), which is above 180 °C when treated at 200 °C. This analysis confirms the successful stabilization of lignin fibers without additives, with a yield of stabilized fibers close to 80%.

**Figure 3:**
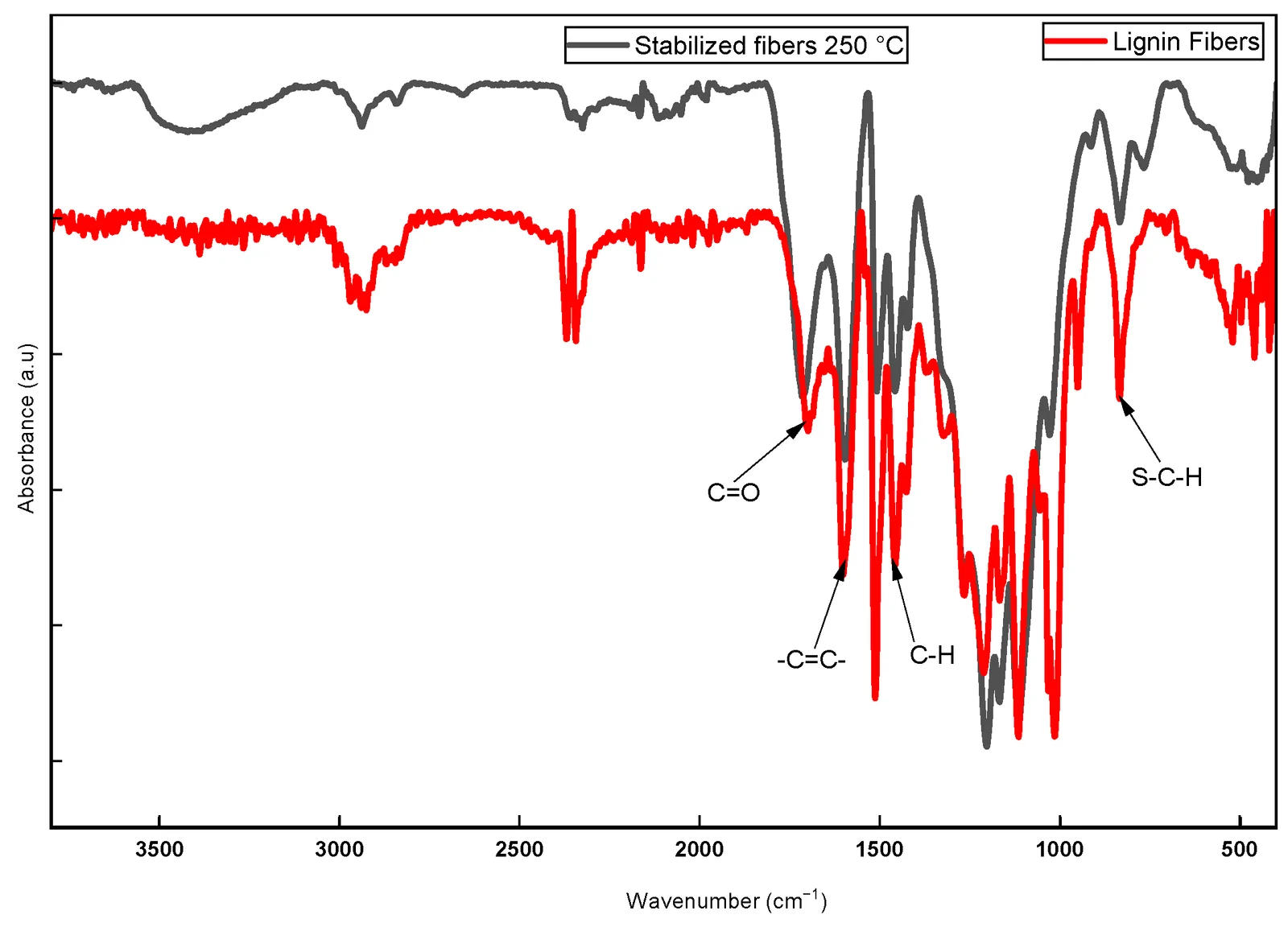
FTIR spectrum of the electrospun and stabilized lignin fibers.

FTIR analysis, which is essential for determining the functional groups of materials, was performed on stabilized and unstabilized lignin fibers (Figure 3). The results show changes in the stabilized fibers. These changes are characterized by the elimination and reduction of certain functional groups [26]. The spectrum of stabilized lignin fibers reveals the main peaks at 1708, 1602, 1457, 1211, 1115 and 833 cm^−1^, reprensenting the fonctiosnal group -C=O, C=C, *sp*^3^ hybridized C-H, -C-O and -C-H from syringyl units respectively, as well as a sulfur containing group at 420 cm^−1^ [19,41,45,46,47,48]. These peaks confirm the presence of both oxygenated and carbon groups, but also a low content of hydrogen and sulfur, which was detected in the electrospun lignin fibers, attesting to the retention of sulfur originating from DMSO. The difference between stabilized and unstabilized lignin fibers is observed in the peaks at 2369 and 2344 cm^−1^ present in the lignin fibers. These peaks are characteristic of O=C=O-type functional groups resulting from the transformation of lignin into lignin fibers during the electrospinning process [26].

An absence or reduction of -CH (2900 cm^−1^) and -OH (3400 cm^−1^) groups—which are abundant in lignins, and particularly in the organosolv lignin of *Miscanthus x giganteus*—is noticeable [26]. This is in agreement with similar studies on electrospun lignins and their stabilization [26,45,48]. The stabilized fibers confirm both the dehydrogenation associated with the stabilization process and the participation of -OH groups in the radical reactions leading to the formation of crosslinked structures, as described in the literature [14,41]. According to [19], during stabilization, the dehydrogenation reaction occurs by breaking up the hydrogen bonds in CH_2_ groups. These dehydrogenation reactions are largely favored by the increase in stabilization temperature and the supply of atmospheric oxygen [18].

Although lignin is primarily composed of aromatic moieties, stabilization promotes cyclization of lignin fibers through the ionization of oxygen containing groups originating from both hydroxide groups (-OH) and the abundant β-O-4 ether bonds [16]. These initially very weak bonds undergo homolytic cleavage, thus promoting the cyclization of the resulting radicals [18]. Based on the functional groups identified in the FTIR spectra, one can speculate that the coupling of radicals from homolytic cleavage of the cited bonds involves ring closures in the absence of hydrogen.

**Figure 4:**
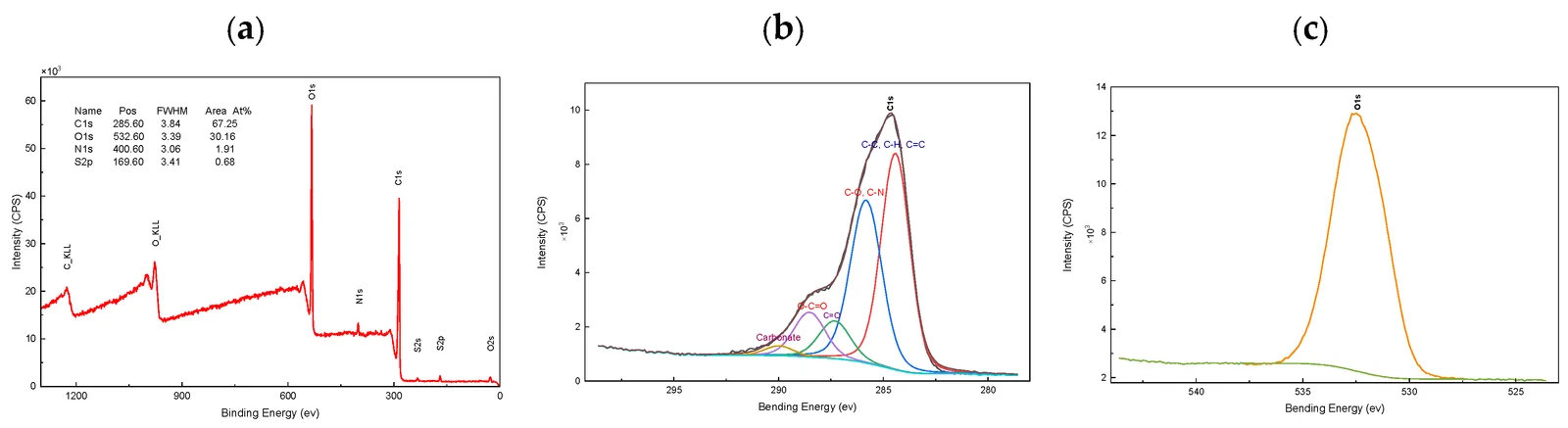
XPS spectra of the stabilized lignin fiber: (**a**) Core spectrum, (**b**) C1s deconvolution, (**c**) O1s deconvolution.

The XPS spectral analysis of the stabilized lignin fibers demonstrated C1s, O1s and S2p peaks and these spectra are shown in Figure 4. As illustrated in Figure 4b,c, the C1s core-level spectra of the stabilized fibers are deconvoluted into four peaks. The main peak is attributed to graphitic carbon at 284.7 eV (C–C), 285.4 eV (C-O), 285.6 eV (C=C) and 289.7 eV (O-C=O) [48].

In the C1s deconvolution spectrum (Figure 4b), the presence of C-C, C=C, and C-O bonds can be distinguished. These different bond types confirm the results of FTIR analysis (Figure 3) and are consistent with the structure of stabilized fibers, which are predominantly composed of carbon and oxygen. For C=C bonds, it is more likely that they are mainly C-C, as the intensity of the C1s peak corresponding to these types of bonds is less than 288.8 eV. According to [41], C=C bonds generally correspond to a peak intensity of 288.8 eV. The prevalence of C-C bonds confirms the oxidative transformation related to the loss of hydrogens upon the establishment of new C-C bonds.

The XPS analysis results of the stabilized fibers indicated a carbon content of 67.25%, and an oxygen content of 30.16%. Compared to lignin fibers, a decrease in carbon content of less than 10% is observed, with an increase in oxygen content of more than 40%, confirming that the stabilization objective was achieved; i.e., increased oxygen content is favorable to further carbonization. As the oxidation temperature rises, the oxygen content also increases, demonstrating that oxygen plays a crucial role in stabilizing the initiation of the cyclization process [19].

A small amount of sulfur was observed in the stabilized fibers. Its origin is from the solvent used (DMSO) in the solubilization of the lignin and has no impact on fiber stabilization. The XPS analysis results (Figure 4) are consistent with the FTIR spectrum (Figure 3), confirming the formation of abundant oxygen groups. In addition to the disappearance of the bump observed in DSC (Figure 1), this result confirms successful stabilization, which is induced by oxidation processes consisting both of the loss of hydrogen and of the integration of oxygen containing functional groups in the stabilized fibers [13,19].

**Figure 5:**
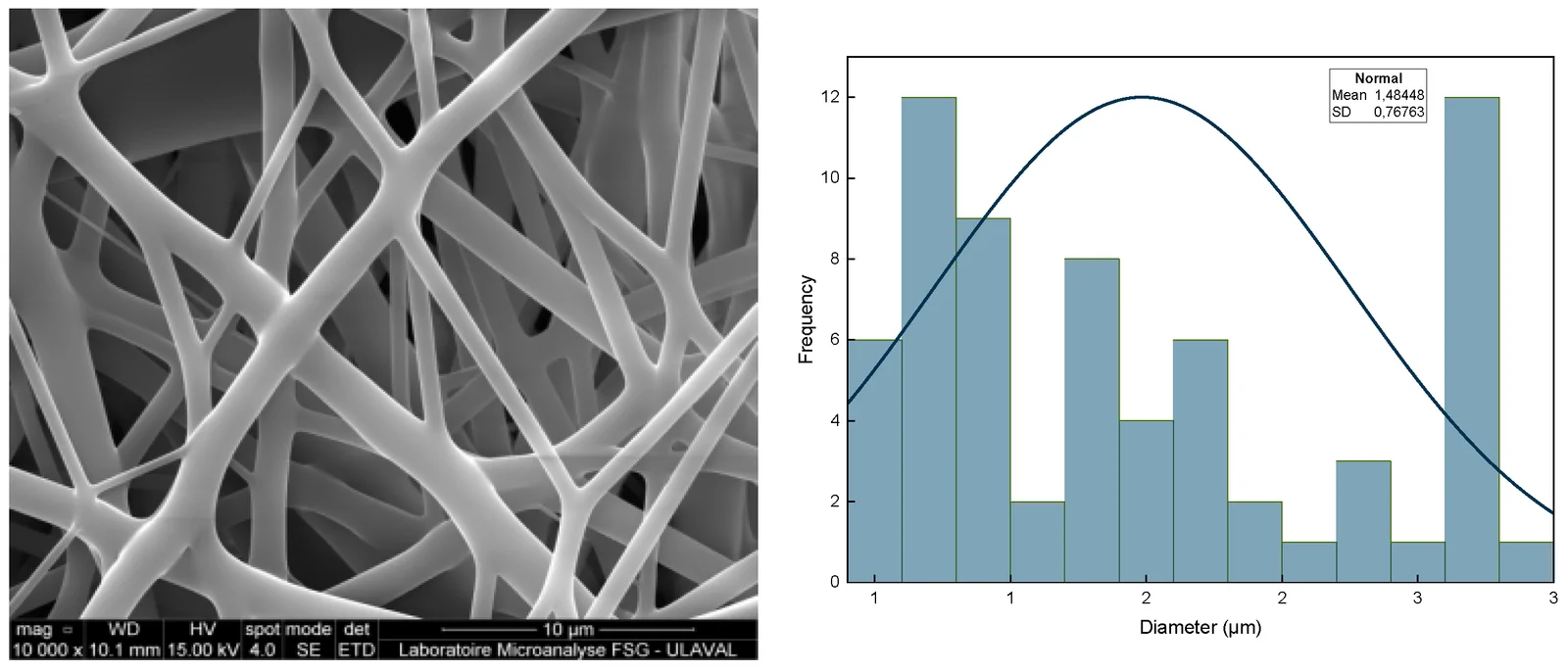
Typical SEM image of the stabilized fibers (**left**), with its corresponding diameter distribution (**right**).

The stabilized fibers were examined by SEM and the obtained micrographs are shown in Figure 5. Morphologically, a variability in the diameter of the stabilized fibers originates from and is closely related to the diameters of the electrospun lignin fibers, rather than from the stabilization process itself. The stabilization process preserved the geometry of the fibers in this study, as the stabilization temperature used was optimized based on the literature on stabilized lignin-based fibers [12,13]. In our previous study [26], significant variability of fiber diameters was observed for electrospun lignin fibers. Compared to unstabilized lignin fibers, a 20% decrease in average diameter compared to the lignin fibers used in this study was determined. This decrease is explained by the loss of material due to stabilization. The decrease in diameter after stabilization is due both to the volatilization of low-molecular-weight compounds and to the disappearance of free volume/porosity resulting from the mobility of molecular chains [41]. Despite an increase in oxygen content in the stabilized fibers, their diameter decreased compared to electrospun lignin fibers [26]. 

Based on the SEM–EDS analysis, comparison of the oxygen content of the fibers before and after stabilization revealed a significant increase of 38%. This indicates that oxygen was incorporated during the stabilization process, which took place under air. Oxygen incorporation is confirmed by the determination of oxygenated functional groups in the fibers [9,13]. According to [33], during lignin stabilization under air, the increase in oxygen content occurs between 200 and 250 °C, mainly via an increase in carbonyl content [33]. Our study shows that fiber stabilization occurs in this temperature range, with a significant oxygen supply. The oxygen supplied during stabilization should allow for the carbonization of the fibers at high temperatures, as it is known to accelerate structural transformations at high temperatures (Table 1) [19]. The EDS analysis results show the change in composition before and after stabilization. This analysis confirms the increase in oxygen and the decrease in sulfur content (Table 1).

**Figure 6:**
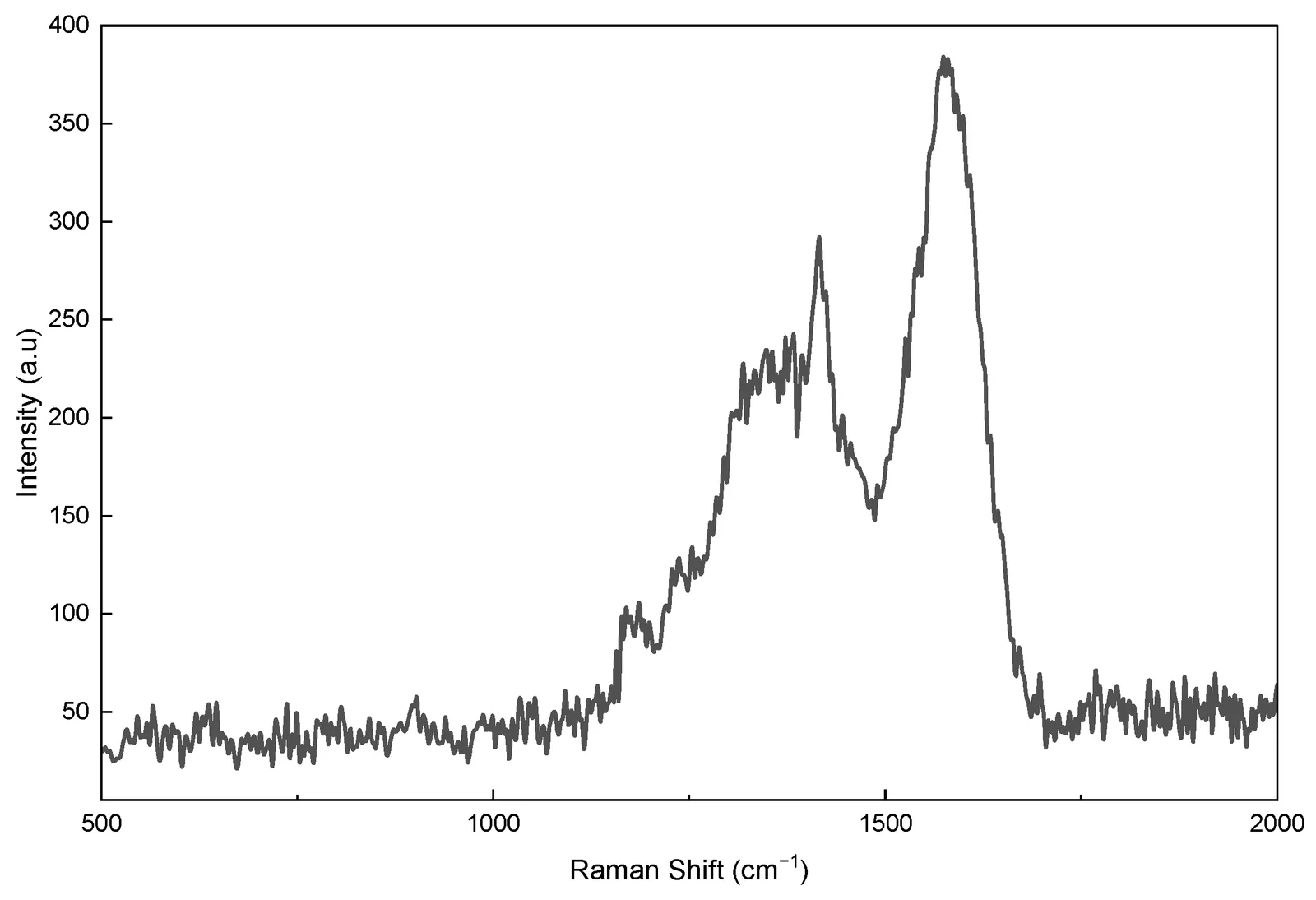
Raman spectrum of stabilized lignin fiber.

To determine the evolution of the transformed material towards a carbonaceous state, Raman spectroscopy analysis was performed on the stabilized fibers (Figure 6). Spectroscopy was used because of its ability to characterize materials based on molecular vibrations. Two main peaks (1416 cm^−1^ and 1581 cm^−1^) can be observed in the Raman spectra. These peaks, close to the values corresponding to the D and G bands of the Raman spectra, are slightly shifted and indicate that the graphitic state has not yet been reached. Based on these results, the stabilized fibers are not yet a truly carbonaceous material, hence the difference in their Raman spectral structures. However, while a transformation of the material towards a crystalline structure is beginning to take place, the amorphous character of the lignin remains dominant [41,49]. Looking at the Raman spectrum of the stabilized fibers, an amorphous structure in full development towards a crystalline state is observed.

An intensity ratio between the D and G bands (I_D_/I_G_), reflecting the degree of carbon disorder, was found to be 0.76 in the stabilized fibers. According to [50], the lower the I_D_/I_G_ value, the fewer defects the graphitic carbon structure exhibits. With a carbon content of 68%, the stabilized fibers are not typically a carbon material. The crystallinity revealed by the I_D_/I_G_ ratio could derive from a stacking of carbon and oxygen atoms favored by radical reactions occurring during stabilization. The I_D_/I_G_ result does not yet show a graphite-like structure as such. In fact, it cannot yet be compared to the ‘main carbon materials’ (carbon black, carbon nanotube, graphene, or graphite); however, it is included here to indicate the tendency of graphitic structure formation.

Despite undergoing graphitization, the process led to material crystallization through the cyclization of the aromatic cores. In fact, since it is rich in syringyl units, the lignin derived from *Miscanthus x giganteus* is less favorable to the condensation reactions of aromatic rings because there are no possible bonds on the C5 carbon. However, cyclization could occur through oxygen atoms introduced during stabilization in the presence of air. According to [5], stabilization around 200–300 °C in an oxidizing environment promotes cyclization reactions in the lignin fibers.

**Figure 7:**
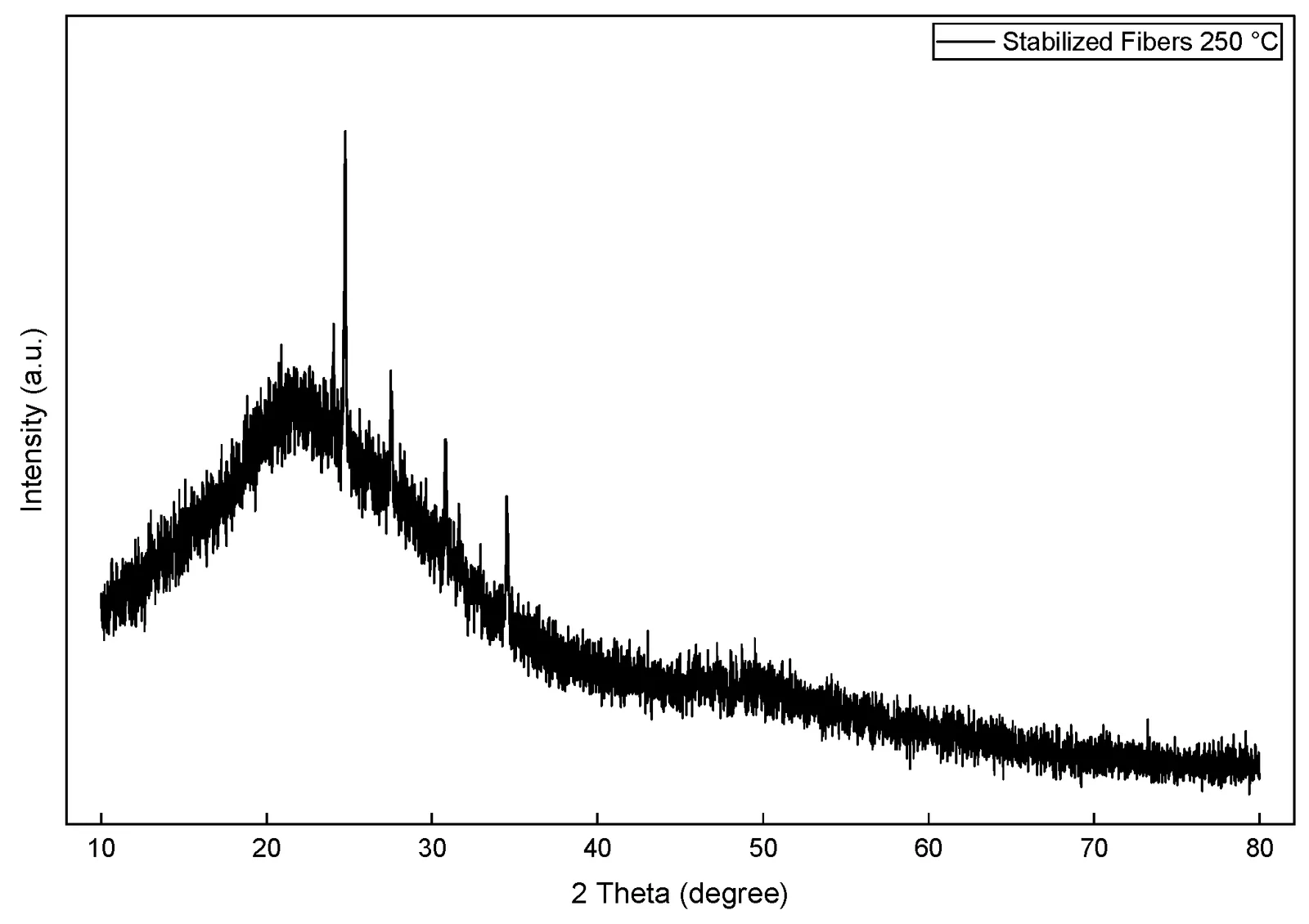
XRD spectrum of the stabilized lignin fibers.

X-ray diffraction (XRD) spectral analysis of the stabilized fibers (Figure 7) reveals a diffraction peak around 2θ ≈ 23°, corresponding to the [002] crystallographic plane of graphitic carbon, consistent with published data [12,41]. The peak width indicates that the material studied is amorphous and contains only a small proportion of crystalline structures. This result is consistent with Raman spectroscopy (Figure 6), which revealed an amorphous structure in the stabilized fibers with cyclization of the aromatic rings, demonstrating progression towards a crystalline state.

**Figure 8:**
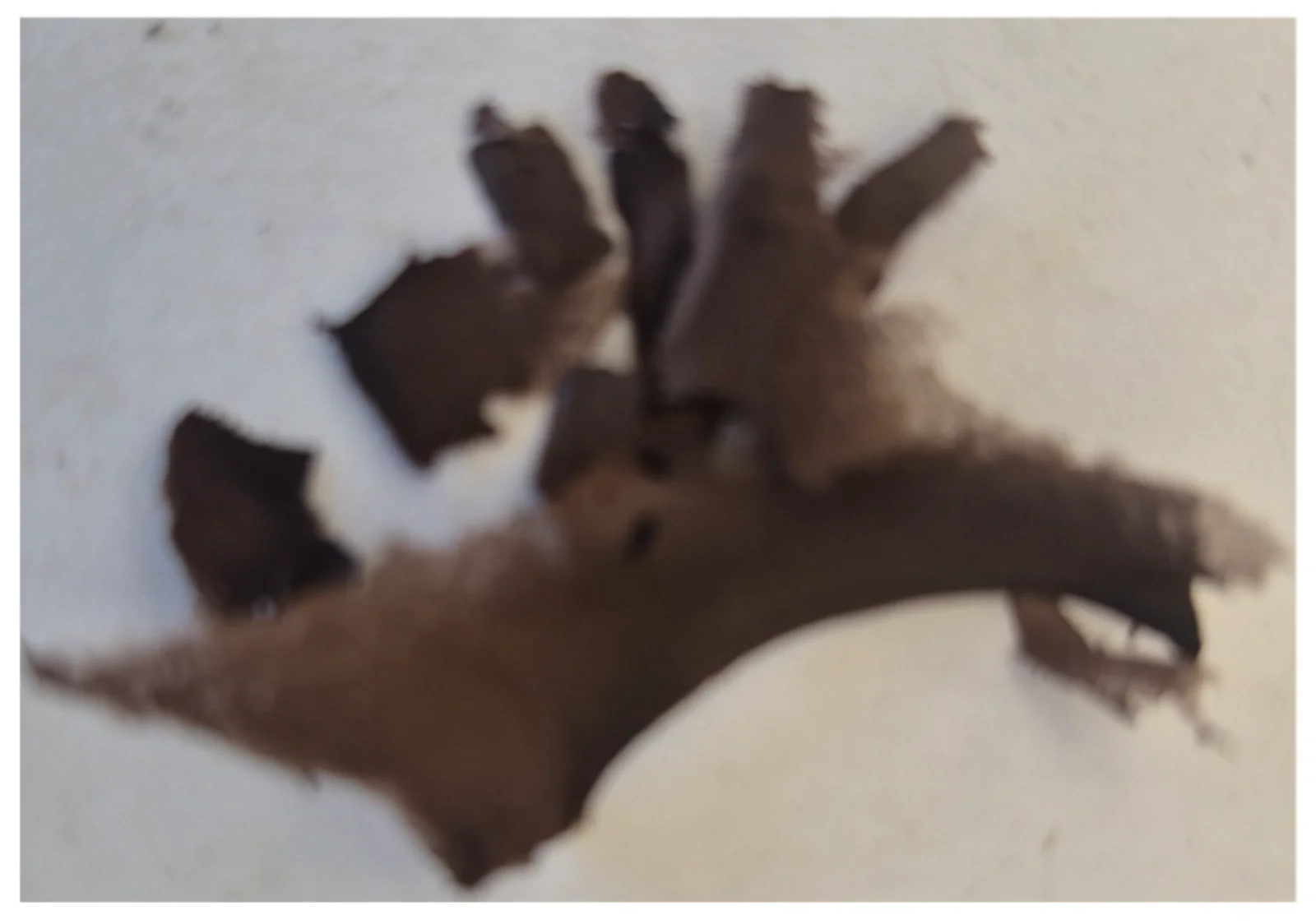
Image of stabilized fibers.

The grain size (*Lc*) was determined from the XRD spectrum analysis of the stabilized fibers and was found to be 0.90 nm. This value is very low, indicating a limited tendency of the material to crystallize at this stage. The evolution of the grain size makes it possible to anticipate how the graphene structure would develop in the material as it is further transformed into carbon fiber [12]. At this stage of the transformation, only a very low-quality graphene-like material is formed. Estimation of the crystalline fraction of the stabilized fibers revealed that it accounted for 38% of the total material; in other words, the majority of the material remains in an amorphous state at this stage. The image in Figure 8 shows the state of the stabilized fibers.

### 3.2. Carbonization of the Stabilized Miscanthus x giganteus Lignin Fibers

**Figure 9:**
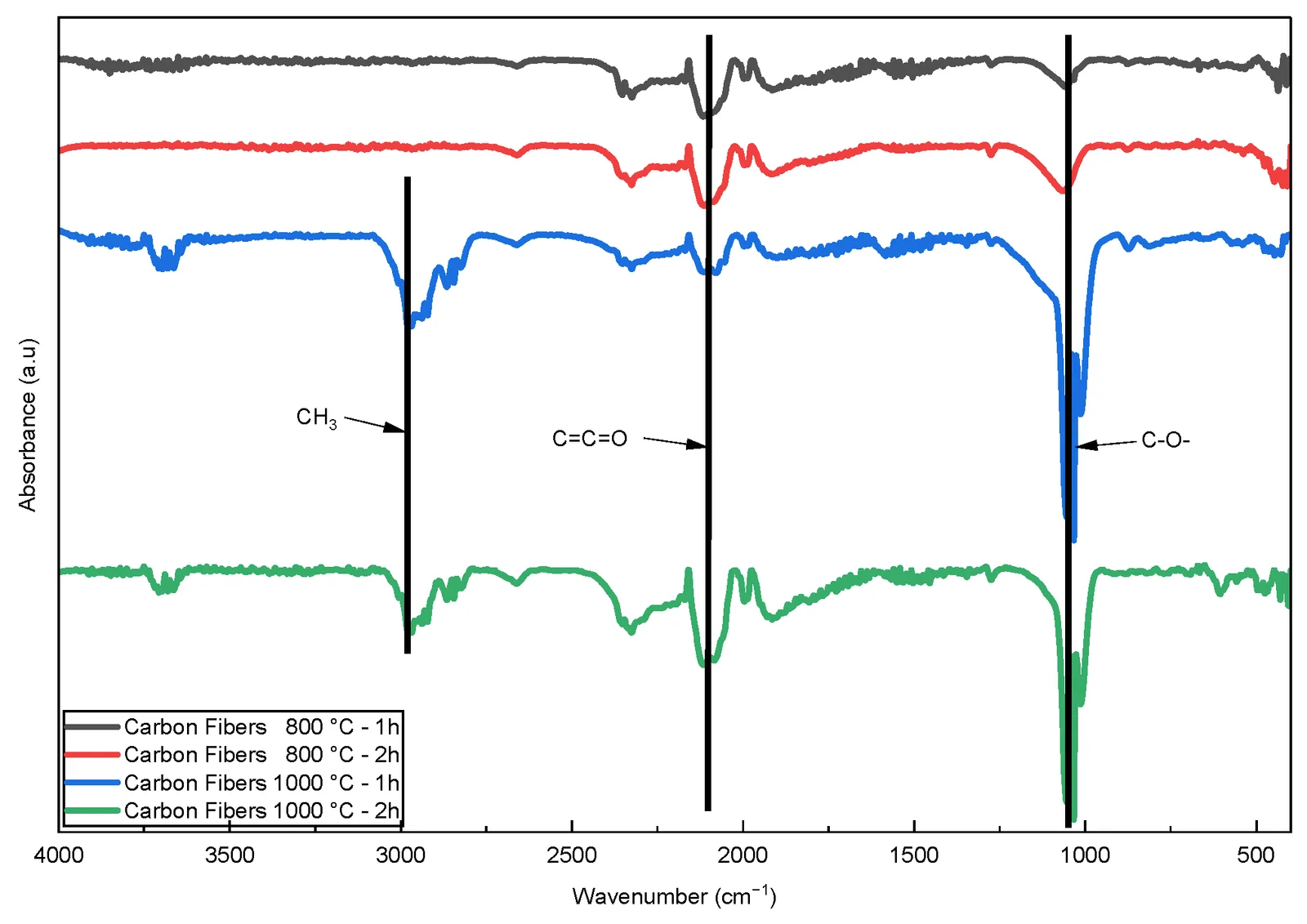
FTIR spectrum of the carbon fibers carbonized under different conditions.

FTIR spectral analysis of the fibers obtained after carbonization of stabilized *Miscanthus x giganteus* lignin fibers under nitrogen, heated at 5 °C/min to maximum temperatures of 800 and 1000 °Cn and held at these temperatures for 1 and 2 h, yielded the results shown in Figure 9. From this analysis, four main peaks were identified, common to all the carbon fiber samples obtained. These peaks, located around 3000, 2150, and 1066 cm^−1^, are characteristic of the functional groups –C–H, C=C–O, and C–O, respectively [19,48]. The peak around of 3000 cm^−1^ was identified only for carbon fibers obtained at 1000 °C. It is characteristic of C–H stretching in the aliphatic groups –CH_3_ and –CH_2_, reflecting the persistence of lignin side chains [1]. The C-H peak reveals a difference in the hybridization state of the carbon atoms between the carbon fibers obtained at 800 and 1000 °C. Despite a small peak indicating a trace of sulfur (around 430 cm^−1^), the resulting CFs are mainly composed of carbon and oxygen. The remaining sulfur content is only 0.20% in CF obtained from electrospun lignin fibers.

Compared to the FTIR for stabilized fibers (Figure 3), a difference is observed with CF between 1200 and 1700 cm^−1^, as these peaks do not appear in the FTIR spectra for carbon fibers (Figure 9). These absorption bands are characteristic of aromatic skeleton vibrations, indicating that the lignin aromatic skeleton in stabilized lignin fibers was retained [1]. The absence of bands between 1200 and 1700 cm^−1^ also implies a low presence of oxygenated functional groups, which are assumed to have been eliminated [3,46]. The decrease in or near absence of functional groups around 1200–1700 cm^−1^ is usually attributed to the elimination of carbon dioxide and carbon monoxide groups during carbonization [13]. A common feature of FTIR spectra of stabilized and carbonized fibers is the absence of a peak around 3400 cm^−1^ which is attributed to the –OH functional group. According to [51], a smaller amount of –OH groups contributes to improved crystallization.

The FTIR spectrum of carbon fibers reveals strong deoxygenation, as evidenced by the difference in peak intensity compared to the FTIR spectrum of stabilized fibers. The decrease in peak intensity associated with the absorption bands of oxygenated groups is attributed to greater deoxygenation and a higher crystalline state of carbon fibers compared to stabilized fibers [9]. This difference in intensity is observed across all peaks, with a more pronounced effect on the peak at 1060 cm^−1^. During the carbonization process, a large part of the oxidized compounds are eliminated through dehydration and decarboxylation reactions, hence the FTIR spectral features of the aforementioned fibers [33]. The analysis of the FTIR spectra shows a tendency for graphite to form in the carbon fibers obtained by heating the stabilized lignin fibers, with changes in the intensity of absorption peaks related to specific functional groups, which vary depending on the process parameters.

**Figure 10:**
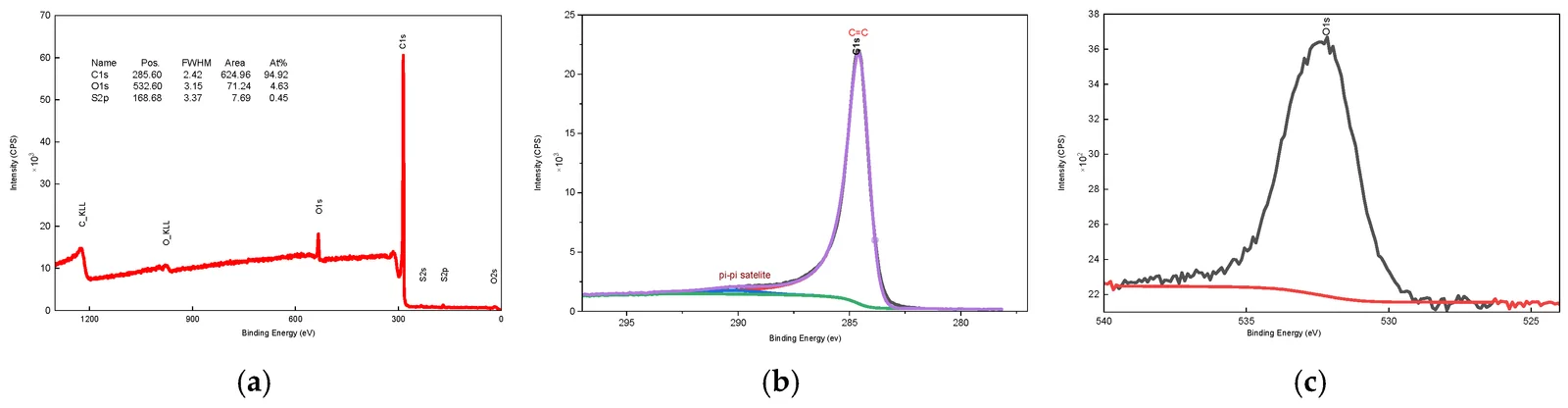
XPS analysis of the carbon fibers produced at 800 °C for 1 h: (**a**) core spectrum, (**b**) C1s deconvolution and (**c**) O1s.

**Figure 11:**
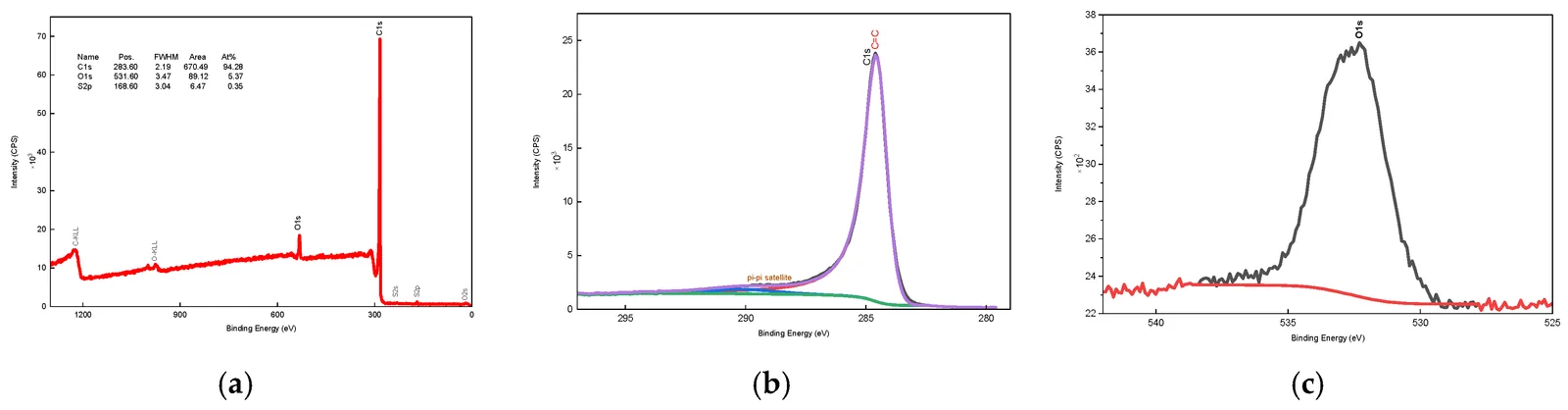
XPS analysis of the carbon fibers produced at 800 °C for 2 h: (**a**) core spectrum, (**b**) C1s deconvolution and (**c**) O1s.

**Figure 12:**
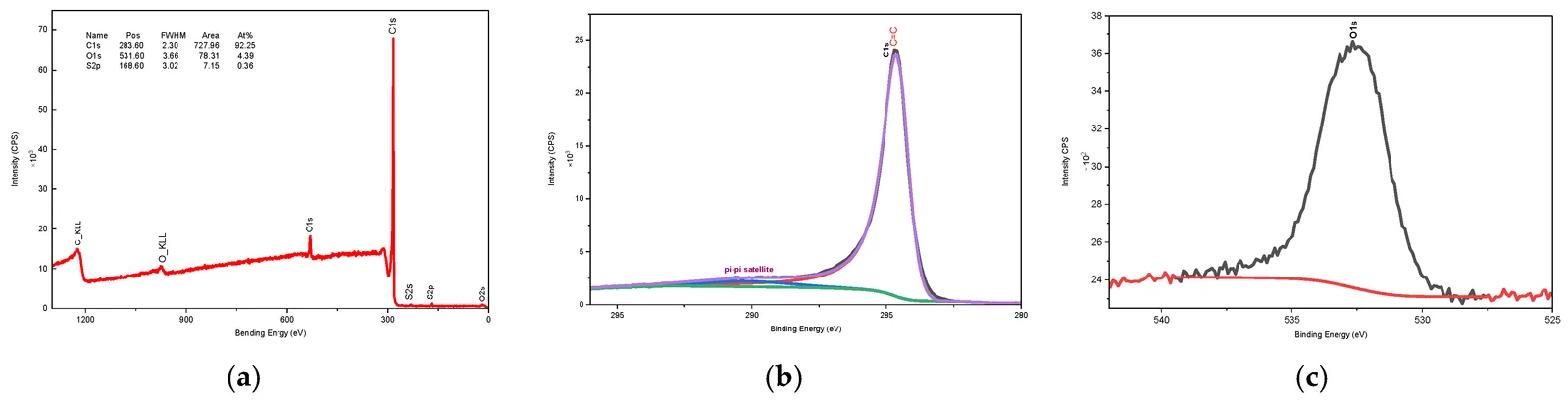
XPS analysis of the carbon fibers produced at 1000 °C for 1 h: (**a**) core spectrum, (**b**) C1s deconvolution and (**c**) O1s.

**Figure 13:**
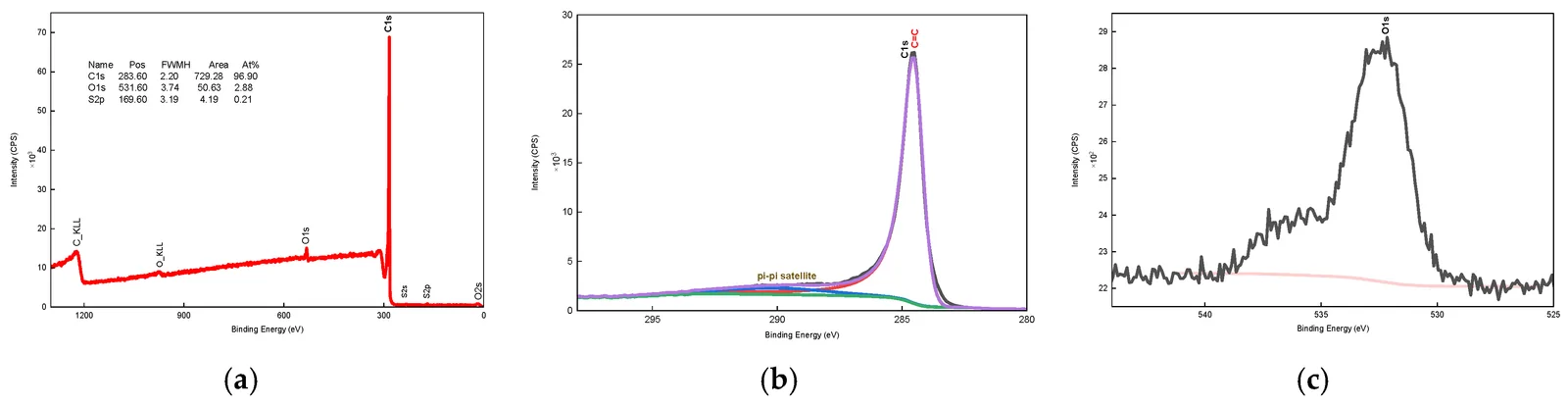
XPS analysis of the carbon fibers produced at 1000 °C for 2 h: (**a**) core spectrum, (**b**) C1s and (**c**) O1s.

The analyses of XPS spectra for the CF gave the results shown in Figure 10, Figure 11, Figure 12 and Figure 13. The full XPS spectra of the carbonized samples revealed the presence of carbon, oxygen, and sulfur within the resulting CF (Table 1), confirming the results obtained by the FTIR analysis (Figure 9). As illustrated in Figure 10, Figure 11, Figure 12 and Figure 13, the C1s core spectrum of different samples is deconvoluted into a single peak. This peak is attributed to graphitic carbon at 285.60 eV (C=C), as reported in the literature [48]. The *sp2* hybridized C=C double bond is proposed to be implemented in a graphitic material, as followed by Raman spectral studies [52]. Oxygen and trace amounts of sulfur from the use of DMSO remain in all studied materials: electrospun lignin, as well as stabilized and carbon fibers, with a decreasing trend as carbonization conditions became more intensive (Table 2). Oxygen is detected at 531.60 eV, indicating C=O-type functionalities [48]. The XPS analysis results show a very small difference in the spectra between 800 and 1000 °C; i.e., the nature of the material obtained at the end of carbonization is similar. These results are consistent with observations from FTIR spectra (Figure 9).

The XPS results confirm the presence of carbon, oxygen and sulfur in organosolv lignin-based CF produced in this study. The carbon fiber obtained at 800 °C contains 94.92% carbon, 4.63% oxygen, and less than 1% sulfur. As the temperature increased to 1000 °C, the carbon content reached 97%. Compared to stabilized fibers, a 40% increase in carbon content is obtained. This indicates a significant removal of oxygen resulting from both the dismutation reactions yielding carbon dioxide and from the removal of volatile matter [18,33]. During carbonization, a high amount of the oxygen incorporated into the fibers during stabilization is released via carbon monoxide and carbon dioxide, as reported in previous studies [13,18].

The evolution of carbon content in the fibers is presented in Table 2. The value varies slightly depending on the temperature and time imposed. The increase in carbon content as a function of temperature or time was 5% between 800 and 1000 °C for treatment times of 1 and 2 h. The carbon content determined by elemental analysis increases with treatment temperature, consistent with the data presented in the literature [18]. In this study, starting at 800 °C, a very slight change in carbon content was observed, which suggests stable carbonization in the temperature range explored here. According to [53], the carbon content of the material is almost constant around 900 °C. A value of 97%, as determined here for CF produced at 1000 °C, is higher than the average carbon content reported for CF of various origins on the market [2].

**Figure 14:**
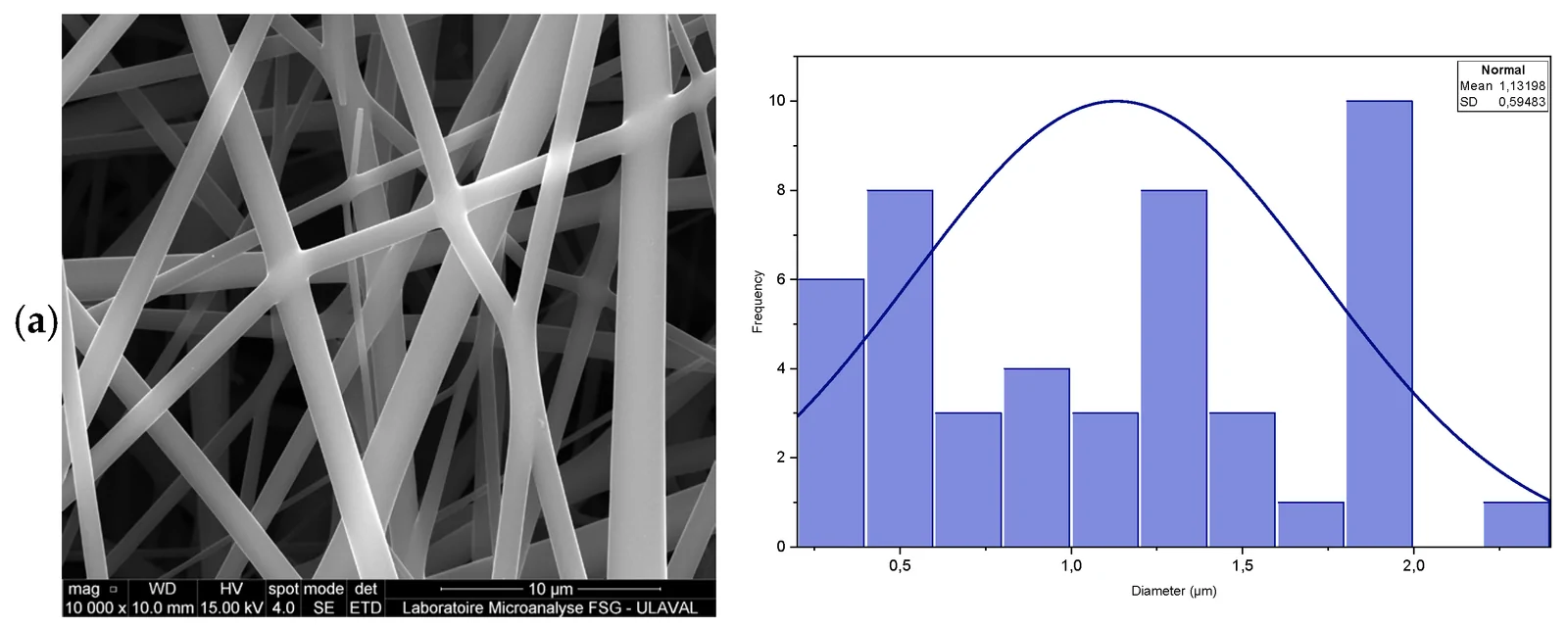

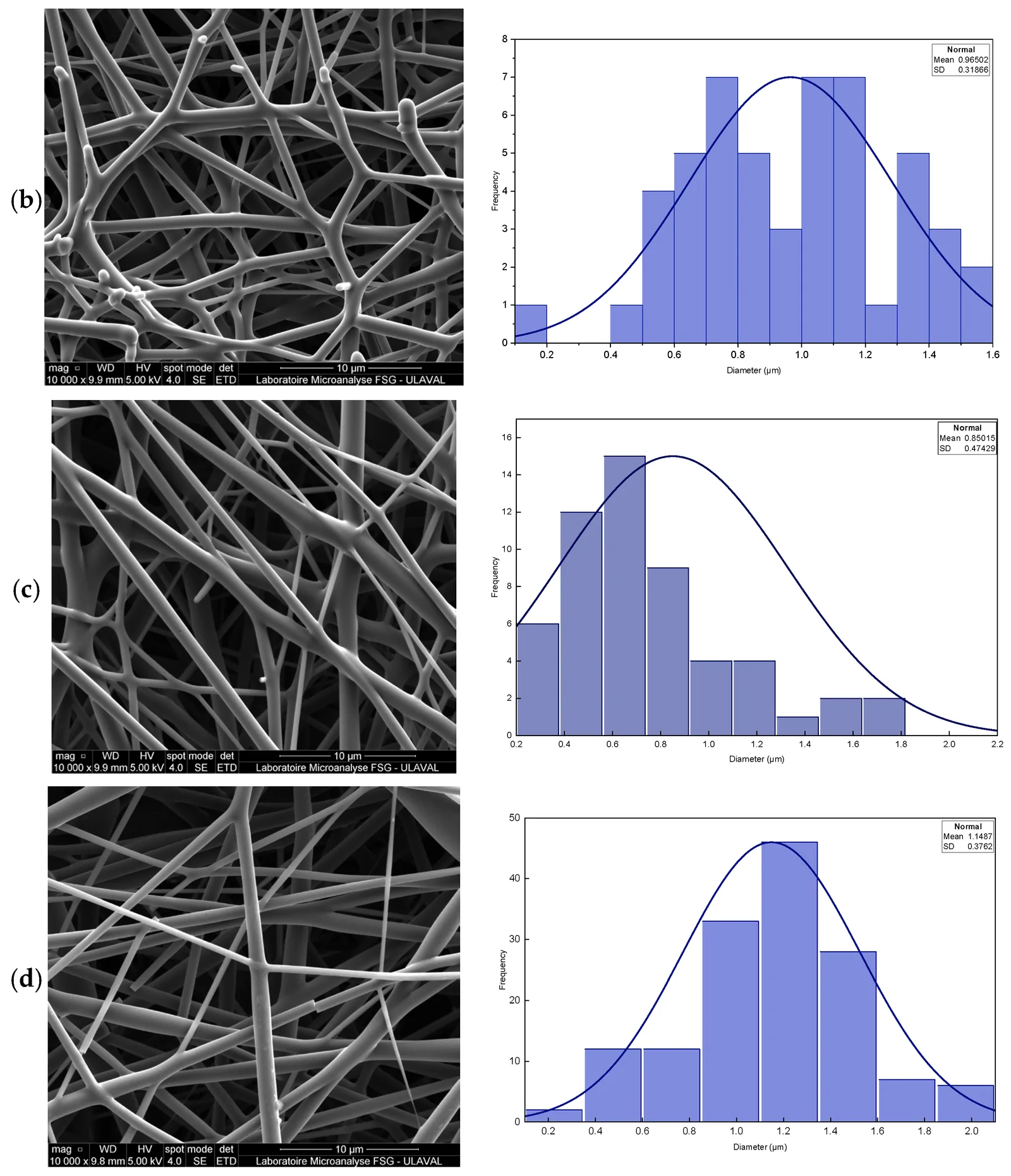
SEM analysis (images on the left and diameter distribution on the right) of CF produced under different conditions: (**a**) 800 °C for 1 h, (**b**) 800 °C for 2 h, (**c**) 1000 °C for 1 h and (**d**) 1000 °C for 2 h.

The results of the SEM analysis of the carbon fibers obtained at different temperatures are presented in Figure 14. This analysis reveals the evolution of fiber properties, both in terms of morphology and diameter. The SEM images show well-separated fibers with submicrometer diameters and nearly cylindrical shapes. For fibers produced at 800 °C, an average diameter reduction of 20% is observed. A morphological difference is also observed in the carbon fibers obtained at 800 °C between 1 and 2 h. After 2 h, the fibers exhibit a more or less twisted structure. According to [34], carbonization of lignin-based fibers is a process involving heat and mass transfer due to the high temperature applied, resulting in the preferential removal of non-carbon elements from the fibers and a decrease in their diameter [34].

The incubation time appears to have an impact on the morphology of the resulting carbon fibers, as observed in this study. Carbon fibers produced at 1000 °C show an average reduction in fiber diameter of about 30% compared to the starting electrospun lignin fibers. Carbon fibers obtained after 1 or 2 h at the same maximum temperature of carbonization showed limited variation in diameter or morphology. The diameter appears to stabilize and does not vary significantly between 1 and 2 h. This near-constant diameter is attributed to the elimination of the majority of the volatile compounds subjected to carbonization [53]. The low molar mass oxygen containing molecules have left during carbonization, leaving behind almost pure carbon, the fine structure of which is of interest for this study. Overall, a decrease in fiber diameter during carbonization is determined.

**Figure 15:**
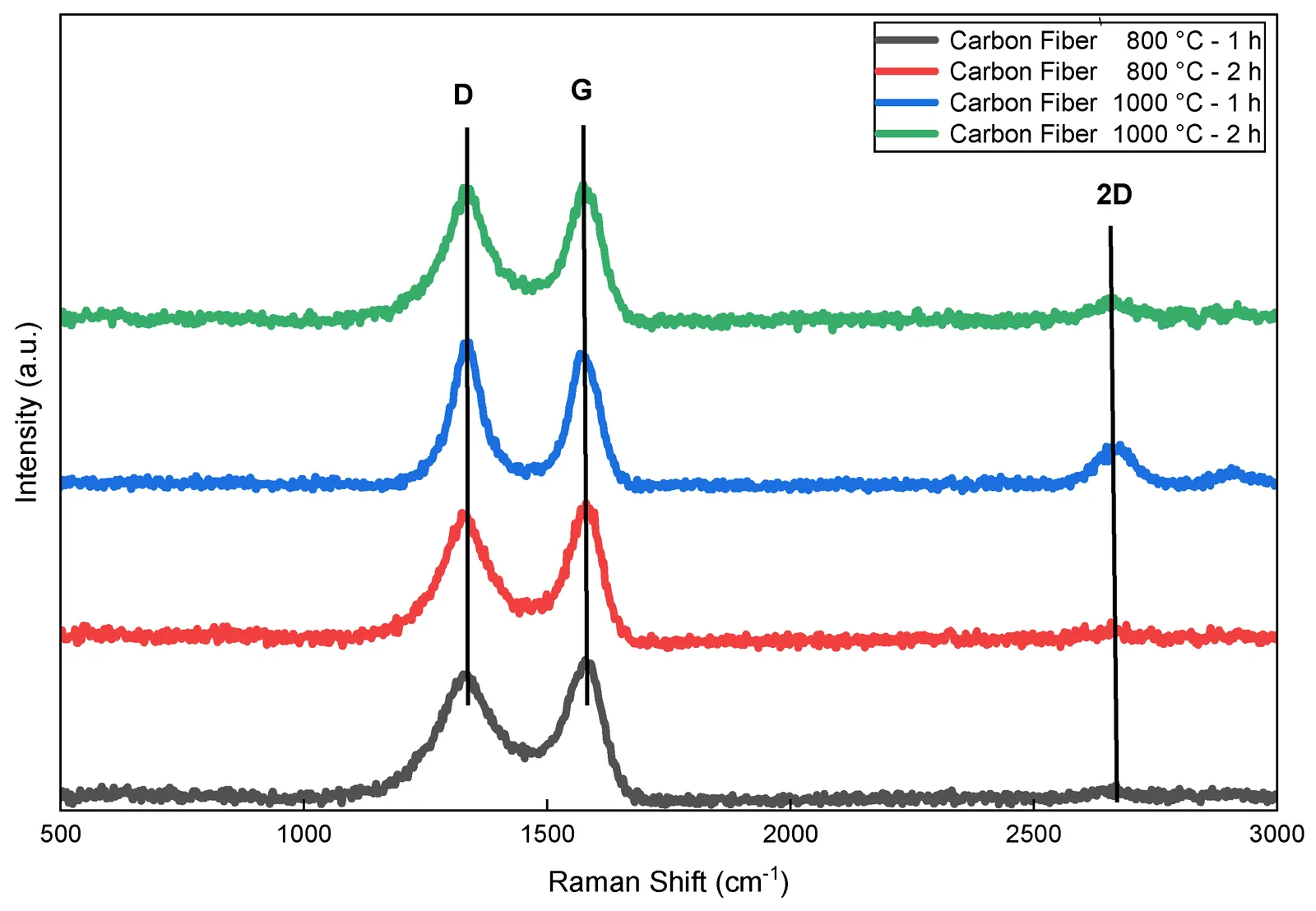
Raman spectra of the carbon fibers produced under different conditions.

Raman spectroscopy analysis, performed on the obtained CF, yielded the results reported in Figure 15. This analysis, which is useful for characterizing carbon-based materials, provided information on their more ordered state and partially crystalline nature. The Raman spectra show the typical characteristics of carbon materials, with the presence of two distinct Raman bands at 1350 cm^−1^ (presence of disordered carbon, D band) and 1590 cm^−1^ (presence of ordered carbon, G band) for CF obtained at 800 °C [16,21,41,49]. For CF obtained at 1000 °C, the Raman spectra exhibit a small third 2D peak symmetric to D. By calculating the intensity ratio of the D and G bands (R = I_D_/I_G_), the results give 0.91, 0.93, 1.07, and 0.97, respectively, for CF obtained at 800 °C for 1 h, 800 °C for 2 h, 1000 °C for 1 h and 1000 °C for 2 h (Table 3). According to [52], the spectral line shape of carbonaceous materials changes radically depending on the excitation wavelength, and this dependence can serve as a unique signature for identifying the type of carbonaceous material. When using a laser with a wavelength of 532 nm, the Raman spectrum with a D peak intensity lower than the G peak is recorded, which is consistent with the graphitic structure of carbonaceous materials [11,50].

Based on the results obtained, the CFs produced at 800 °C have a I_D_/I_G_ ratio below 1 (Table 3), confirming a tendency towards the formation of a carbon material with a more ordered structure approaching the graphitic structure [16,50]. The high ID/IG ratio indicates a significant proportion of disordered material—comparable to carbon black, which exhibits a low degree of crystallinity, an intense D band, and a broad G band, reflecting a disordered turbostratic-like structure. Thus, ref. [54] demonstrated the production of carbon nanofibers exhibiting a pronounced D-band, indicating a high degree of disorder.

In the Raman spectra obtained for carbon fibers produced at 1000 °C, the graphene domains exhibit a structural state influenced by the disordered portion of the material. For these fibers, the ID/IG ratio is close to 1, which is higher than the value obtained for fibers produced at 800 °C. This fact may explain the presence of the 2D peak only in the Raman spectra of CF produced at 1000 °C (Figure 15). The presence of 2D peak seems to indicate the absence of extensive graphitic stacking and the prevalence of defect-rich domains instead of crystalline graphene layers in carbonized materials. Such observations were reported for lignin-derived materials by hydrothermal treatments not exceeding 350 °C [55]. According to [54], which presents the results of a study on cellulose-based carbon fibers, the 2D band appearing around 2700 cm^−1^—depending on the laser excitation wavelength—is linked to the number of graphene layers.

The 2D peak observed for carbon fibers produced at 1000 °C demonstrates that, despite the turbostratic nature associated with the non-crystalline amorphous portion of the material, the resulting fibers show an evolution toward a graphene-like material. According to [20], an increase in carbonization temperature should reduce the ID/IG ratio; the lower the I_D_/I_G_ value, the closer the structure is to that of graphite and the greater the interlayer spacing in carbon materials [9,16,48,50]. In the case of CF obtained at 1000 °C, a non-pure graphene material is obtained with an I_2D_/I_G_ of 0.23. For a monolayer or pure graphene, I_2D_/I_G_ was reported to be greater than 1.6 [56].

The crystallite size (*La*) was determined using the I_D_/I_G_ ratio. Table 3 shows that *La* is higher for CF obtained at 800 °C than for 1000 °C. This appears to stem from the material’s evolution, as Raman spectroscopy analysis, according to ASTM E320 guidelines, reveals the existence of four types of carbonaceous materials: carbon black (amorphous), graphite, monolayer graphene, and carbon nanotubes (CNT). The resulting carbon fibers reveal that at 800 °C—despite exhibiting a significant disordered component—their Raman structure more closely resembles that of carbon nanotubes (CNTs) than carbon black. In contrast, at 1000 °C, the carbonaceous material resembles impure graphene due to the presence of disorder in a turbostratic form [54]. These results demonstrate the material’s evolution during carbonization, leading to the formation of a more ordered crystalline structure within the carbon fibers [57].

**Figure 16:**
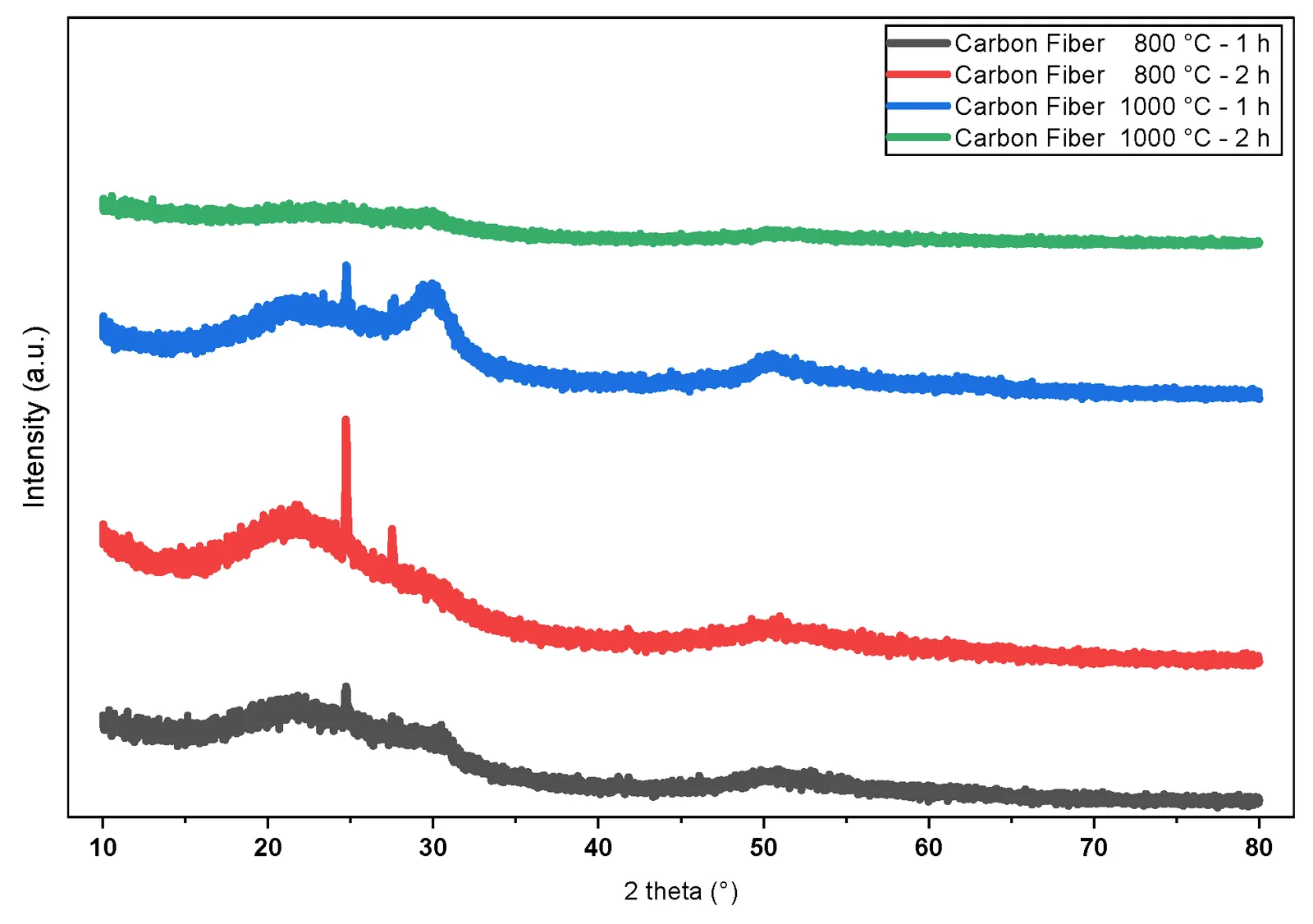
XRD spectra of the carbon fibers produced at 800 °C (1 h and 2 h) and 1000 °C (1 h and 2 h).

Figure 16 presents the X-ray diffraction (XRD) spectra of CF measured parallel and perpendicular to the fiber axis. The results show three broad, low-intensity diffraction peaks appearing at diffraction angles of 23, 31 and 51°. These peaks correspond to the (002), (110), and (004) crystal planes of graphite carbon, respectively, indicating a carbonaceous material with a crystalline structure [12,48,57]. The peak at 31° is primarily observed in the diffractograms of CFs produced at 1000 °C, distinguishing them from fibers obtained at 800 °C. A similar differentiation was observed in Raman spectroscopy analysis between fibers obtained at 800 and 1000 °C, as an additional 2D peak in Raman spectra is present (Figure 15).

For the 2θ peak around 23°, this peak is generally attributed to the crystal size of randomly oriented stacked graphite layers [58]. The intensity and broadening of this peak allowed for the estimation of crystallite size and interplanar spacing. In this study, a broad band is observed in the 2θ (23°) region, characteristic of amorphous carbons, for all the CF samples obtained. The width of the peak around 23° indicates that the carbon nanofibers are primarily composed of an amorphous structure, reflecting a disordered state [15,55].

According to the Scherrer (Equation (1)) and Bragg (Equation (2)) formulas, the crystallite size (*Lc*) and interplanar spacing (d_002_) were quantitatively analyzed to examine the presence of crystalline carbon in the obtained carbon fibers (Table 4). The calculated *Lc* values ranged from 0.95 to 1.99 nm. According to [8], *Lc* values between 0.79 and 1.09 nm correspond to crystals with an average of two to three graphene layers. Despite the predominantly amorphous nature of the material, the obtained *Lc* values indicate the presence of crystalline elements. They indicate the formation of a layered intercalated structure with a long-range structural order within the layer [58]. The obtained d_002_ values are greater than the interplanar spacing of ideal graphite (0.335 nm), indicating the existence of a pre-graphitic turbostratic carbon structure [51].

The size of the crystallites (*Lc*) for CF produced at 800 °C is larger than those obtained at 1000 °C. This result is consistent with Raman analysis (Figure 15) and confirms a phase change from carbon nanotube to graphene. The phase change appears to be favored by the shape or position of the microcrystals. A broadening of the peak in the spectra as a function of increasing temperature is observed. This seems to be confirmed by the results of Raman spectroscopy (Figure 15), which indicates the presence of a graphene-type structure from the 2D peak in Raman spectra obtained for CF produced at 1000 °C. The CFs produced at the same temperature seem to exhibit a change in microcrystalline size over time. It is more advantageous to subject the fibers to carbonization for 2 h at maximum temperature rather than for 1 h.

According to [58], temperature-induced structural ordering favors the reduction of peak broadening and their shift in diffraction patterns towards the values of the graphite phase. The change in state of the material, from carbon nanotube to graphene, does not appear to produce the same results. The broad X-ray diffraction (XRD) peaks (Figure 16) obtained for CF generally indicate a reduced number of crystalline domains, crystalline structures with more defects, or smaller crystallites [9]. As shown in Table 4, the d_002_ spacing is determined to be around 0.44 nm. These samples exhibit an almost constant interplanar spacing, except for the sample produced at 800 °C for 2 h. Similar results have been reported regarding the effect of adding acetylated kraft lignin to PAN in the production of CF [3].

**Figure 17:**
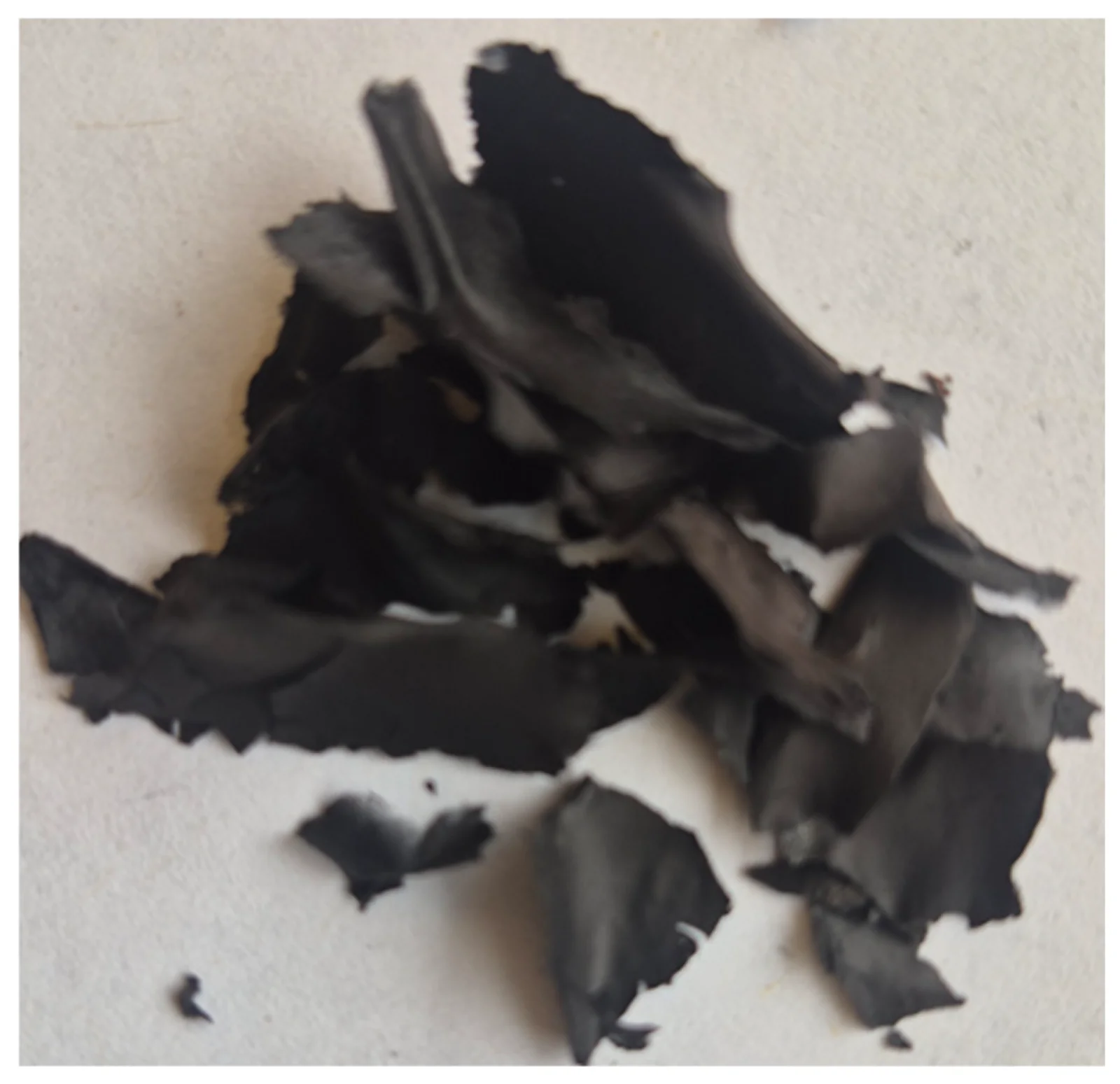
Image of stabilized lignin-based fibers after carbonization at 1000 °C.

The evolution of carbonaceous materials obtained after carbonization at 800 and 1000 °C appears to reveal apparent physical changes. In particular, a third peak (2D) appears in the Raman spectra (Figure 15) and intensifies with increasing carbonization temperature. This 2D peak, which is related to graphene-type materials, becomes more pronounced for CF produced after carbonization under nitrogen for 2 h at 1000 °C. Curiously, the XRD spectrum obtained for that same sample exhibits an additional peak, indicating a physical change that occurs when the temperature is raised to the highest level. One could speculate that the high-temperature treatment applied during the carbonization step caused the “detachment” of a graphene layer from graphitic material, as confirmed by the presence of the peak at 2700 cm^−1^ which is a characteristic peak in Raman spectra of graphene-like materials [54]. Figure 17 shows the aspect of the obtained carbon fibers.

**Figure 18:**
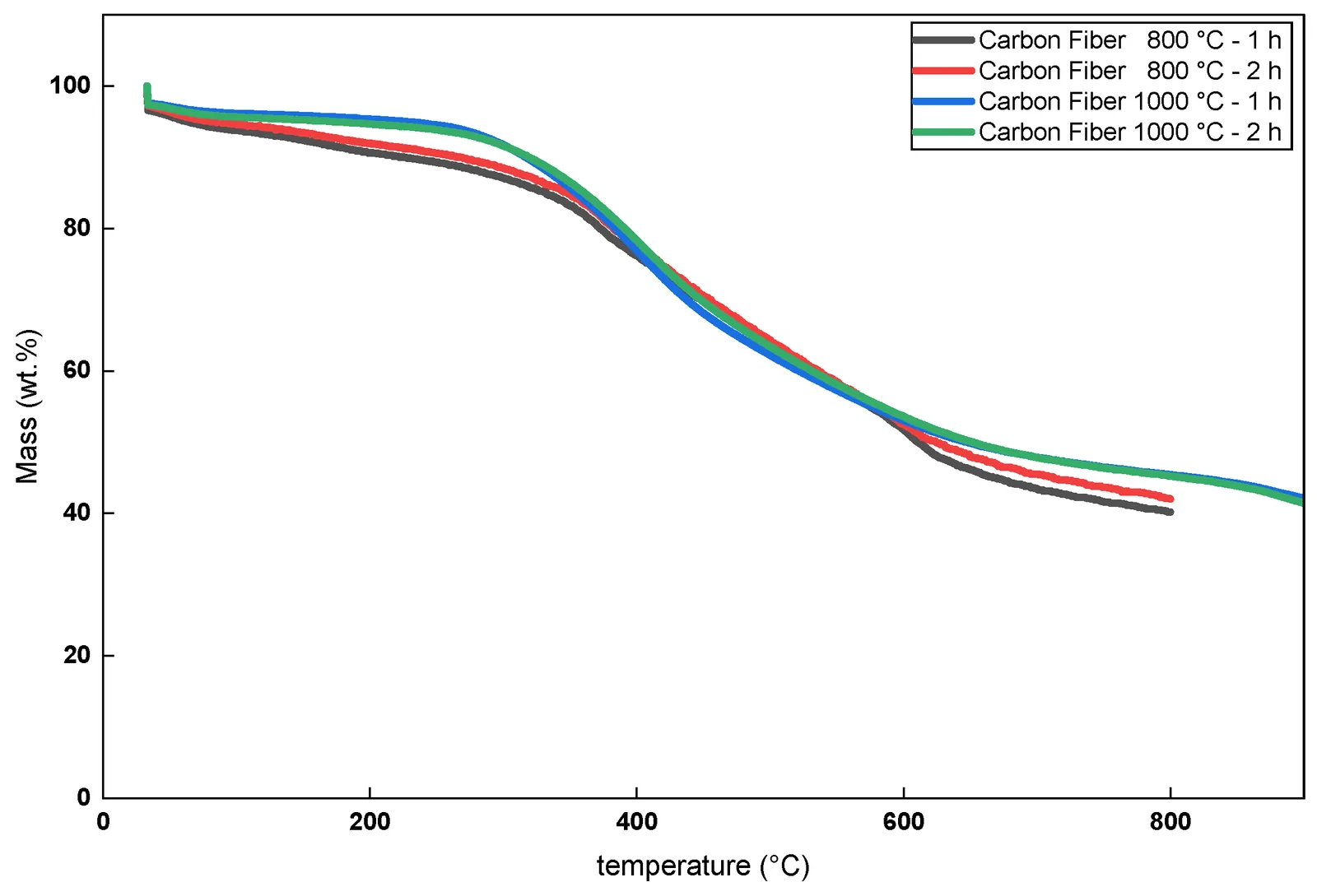
TGA results used to determine the yield of carbon fibers produced by carbonizing stabilized fibers under nitrogen at 1000 °C.

To determine the yield of the obtained CF, a TGA analysis was performed on the stabilized fibers under the experimental conditions used to produce them (Figure 18). This analysis shows a conversion of the stabilized fibers into CF, with a yield of around 40%, depending on the operating conditions. This yield is close to the values reported in the literature for lignin-based CF [2,14,18,53,57,59]. Despite a high stabilization yield, values significantly below 50% of the initial amount of material are obtained for the carbonization step.

The reduction in carbonization yield is explained by the elimination of non-carbon elements. Compared to any other biopolymer in the search for a PAN substitute, lignin can achieve the highest possible carbon yield [10]. The yields, which are virtually identical between 800 and 1000 °C, confirm the results of CF composition analyses performed using XPS and EDX. These analyses showed limited variation in CF composition between 800 and 1000 °C. According to [18], carbonization yields decrease with increasing temperature. The TGA curves of CF obtained at 1000 °C show a continuous decline beyond 800 °C. Considering the TGA curve of CF, significant loss of material occurs between 400 and 600 °C. Reference [53] explained this phenomenon by a devolatilization of lignin at these temperatures.

## 4. Conclusions

This study confirmed that additive-free electrospun Miscanthus organosolv lignin fibers can be stabilized without fusing, while retaining the fibrous morphology during carbonization, and form carbon-rich fibers exhibiting increasingly ordered carbon domains. The stabilization of lignin fibers was achieved by optimizing the operating conditions. Starting from a glass transition temperature (Tg) of 75 °C, it was possible to stabilize electrospun lignin fibers under operating conditions of 0.25 °C/min for a temperature between 200 and 300 °C for 2 h. Stabilizing the fibers resulted in a reduction of 35% in the diameter of the lignin fibers and a loss of material of 20%. Thanks to its structure, which is similar to that of lignin, and the stabilization conditions used, the material obtained without melting was suitable for the subsequent carbonization step.

The carbonization step yielded an evolving material exhibiting two structures: a more ordered carbonaceous structure and the emergence of a graphene-like structure [TS1.1], as evidenced by the appearance of a 2D peak at 2700 cm^−1^ indicating the evolution of the carbonized material towards a higher order. These observations regarding the evolution of the ordered structures upon thermal treatment confirm that the carbon material obtained from lignin only can be refined for improved properties, which should be the subject of further studies.

Future developments involving composites based on this material will demonstrate the application prospects for the biobased fiber currently under development. At this stage, we have successfully produced a biobased fiber with an overall yield of 40%. The fiber’s properties will confirm its potential and future commercial applications once industrial-scale production is achieved.

## Figures and Tables

**Table 1:** EDS analysis results for stabilized and non-stabilized fibers.

Element	Lignin Fiber Composition (Atomic %)	Stabilized Fiber Composition (Atomic %)
Carbon	70.60	64.69
Oxygen	28.07	35.31
Sulfur	1.87	-

**Table 2:** Evolution of the carbon fiber composition as a function of temperature and time.

Conditions	C_XPS_ (Atomic %)	O_XPS_ (Atomic %)	S_XPS_ (Atomic %)
Carbon fibers 800 °C—1 h	94.92	4.63	0.45
Carbon fibers 800 °C—2 h	94.28	5.37	0.35
Carbon fibers 1000 °C—1 h	95.25	4.39	0.36
Carbon fibers 1000° C—2 h	96.90	2.88	0.21

**Table 3:** Results of Raman spectroscopy analysis on the carbon fibers.

Conditions	I_D_/I_G_	*La* (nm)
800 °C—1 h	0.91	4.84
800 °C—2 h	0.93	4.73
1000 °C—1 h	1.07	4.11
1000 °C—2 h	0.97	4.54

**Table 4:** Microcrystalline size and peak broadening at 23 °C for carbon fibers produced under different conditions.

Conditions	*Lc* (nm)	*d*_002_ (nm)
800 °C—1 h	1.99	0.43
800 °C—2 h	1.31	0.47
1000 °C—1 h	1.14	0.44
1000 °C—2 h	0.95	0.44

## Data Availability

The data are available on the hard drives of computers and equipment used at Université Laval.
